# Crystal structure of *Pseudomonas aeruginosa* FabB C161A, a template for structure-based design for new antibiotics

**DOI:** 10.12688/f1000research.74018.1

**Published:** 2021-11-01

**Authors:** Vladyslav Yadrykhins'ky, Charis Georgiou, Ruth Brenk

**Affiliations:** 1 Department of Biomedicine, University of Bergen, Bergen, 5020, Norway

**Keywords:** crystal structure, 3-oxoacyl-[acyl-carrier-protein] synthase 1, FabB, antibiotics

## Abstract

**Background**: FabB (3-oxoacyl-[acyl-carrier-protein] synthase 1) is part of the fatty acid synthesis II pathway found in bacteria and a potential target for antibiotics. The enzyme catalyses the Claisen condensation of malonyl-ACP (acyl carrier protein) with acyl-ACP via an acyl intermediate. Here, we report the crystal structure of the intermediate-mimicking *Pseudomonas aeruginosa *FabB ( *Pa*FabB) C161A variant.

**Methods**: His-tagged *Pa*FabB C161A was expressed in *E.coli *Rosetta DE3 pLysS cells, cleaved by TEV protease and purified using affinity and size exclusion chromatography. Commercial screens were used to identify suitable crystallization conditions which were subsequently improved to obtain well diffracting crystals.

**Results**: We developed a robust and efficient system for recombinant expression of *Pa*FabB C161A. Conditions to obtain well diffracting crystals were established. The crystal structure of *Pa*FabB C161A was solved by molecular replacement at 1.3 Å resolution.

**Conclusions**: The *Pa*FabB C161A crystal structure can be used as a template to facilitate the design of FabB inhibitors.

## Introduction

**Figure 1.**
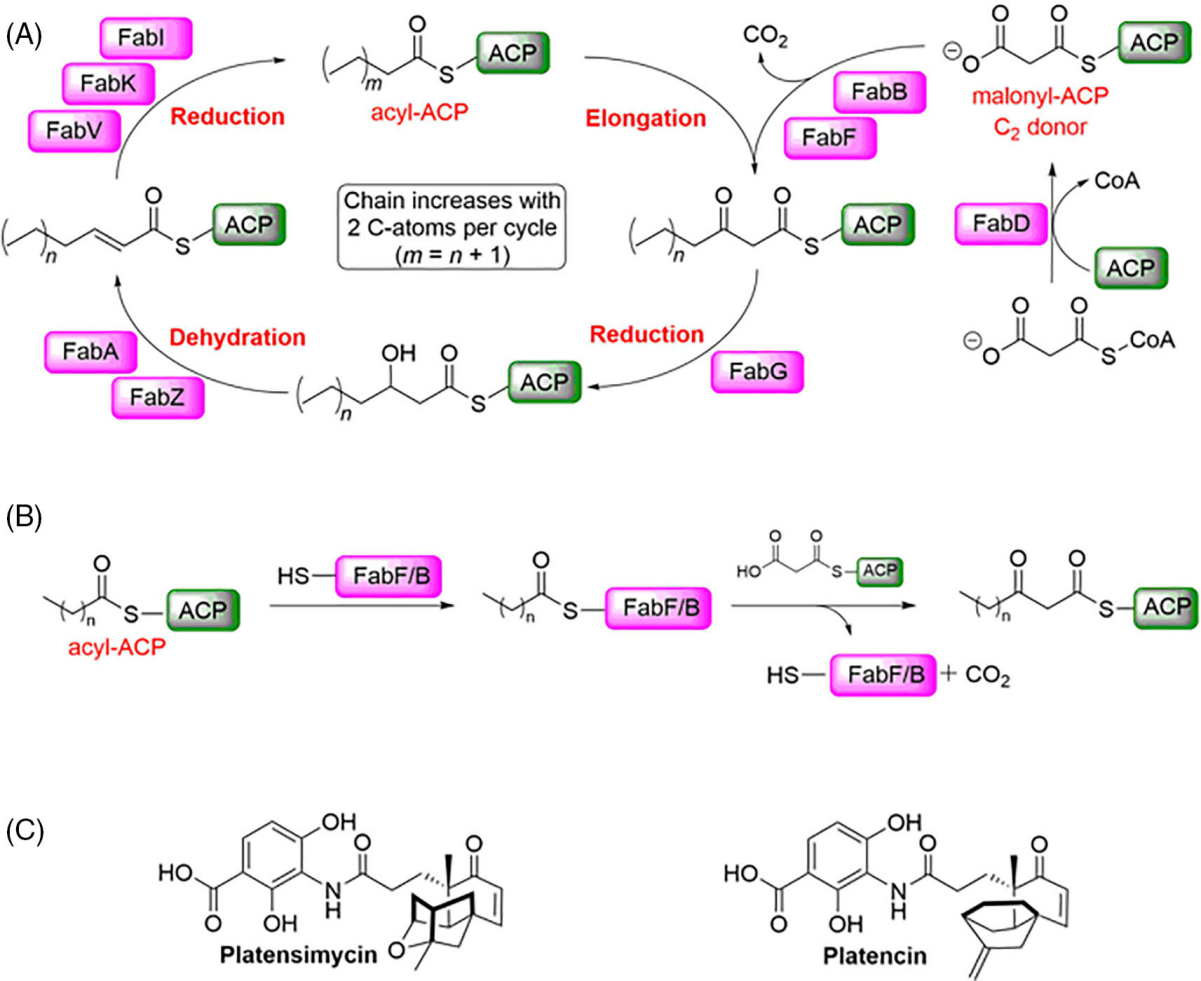
FAS II pathway and its inhibitors. A) Schematic overview of the elongation part of the FAS II pathway. B) Condensation reaction catalysed by FabF/B. (ACP: acyl carrier protein). C) Platensimycin and platencin have been reported as dual FabF/B inhibitors.

New antibiotics are urgently needed to maintain the high standard of living that we have got accustomed to as the antibiotics of today are losing effectiveness faster than they are being replaced by new treatment options. ^1^ If no action is taken, by 2050 drug-resistant infections will kill 10 million people a year worldwide, more than currently die from cancer. ^2^ A possible source for new targets for antibiotics is the fatty acid synthesis (FAS II) pathway ( Figure 1A). ^3^ In this pathway, fatty acid synthesis is carried out by a series of monofunctional enzymes which are highly conserved among microbial pathogens. Genes coding for enzymes in the FAS II pathway have been found to be essential for *P. aeruginosa* in several genetic screens, including the gene for FabB (3-oxoacyl-[acyl-carrier-protein] synthase 1). ^4^^–^^8^

Both, FabB and FabF (3-oxoacyl-[acyl-carrier-protein] synthase 2) catalyse the Claisen condensation of malonyl-ACP (acyl carrier protein) with acyl-ACP ( Figure 1B), but differ in substrate specificity for the fatty acid chain. ^3^ Platensimycin and platencin ( Figure 1C) have been reported to be FabF and FabB inhibitors. ^9^^,^^10^ However, it has been shown that these compounds do not bind potently to the w. t. enzyme, but only to the lauroyl-FabF intermediate ( Figure 1B) and to intermediate-mimicking FabF variants in which the active site Cys has been changed to either Gln or Ala. ^9^^,^^11^ It has been assumed that the same is the case for FabB.

To facilitate structure-based design of FAS II inhibitors, knowledge of the structures in this pathway is essential. Recently, we have reported the crystal structure of *Pa*FabF and the reaction intermediate-mimicking variant *Pa*FabF C164Q. ^12^ Here, we report the crystal structure of *Pa*FabB C161A at 1.3 Å resolution.

## Results

### Protein expression and purification

The gene coding for *P. aeruginosa* PA14 FabB C161A was synthesised and cloned in a bacterial plasmid pET-28a(+)-TEV vector after a DNA sequence coding for a 6-His-tag followed by a TEV cleavage site. To find good expression conditions, seven widely used *E. coli* strains were transformed with the plasmid (BL21 (DE3), BL21 (DE3) pLysS, C41 (DE3), C41 (DE3) pLysS, C43 (DE3), C43 (DE3) pLysS and Rosetta (DE3) pLysS) and screened for protein expression. The best results were obtained with Rosetta (DE3) pLysS cells (data not shown). Therefore, this cell line was used for all subsequent protein expression experiments. ^21^

**Figure 2.**
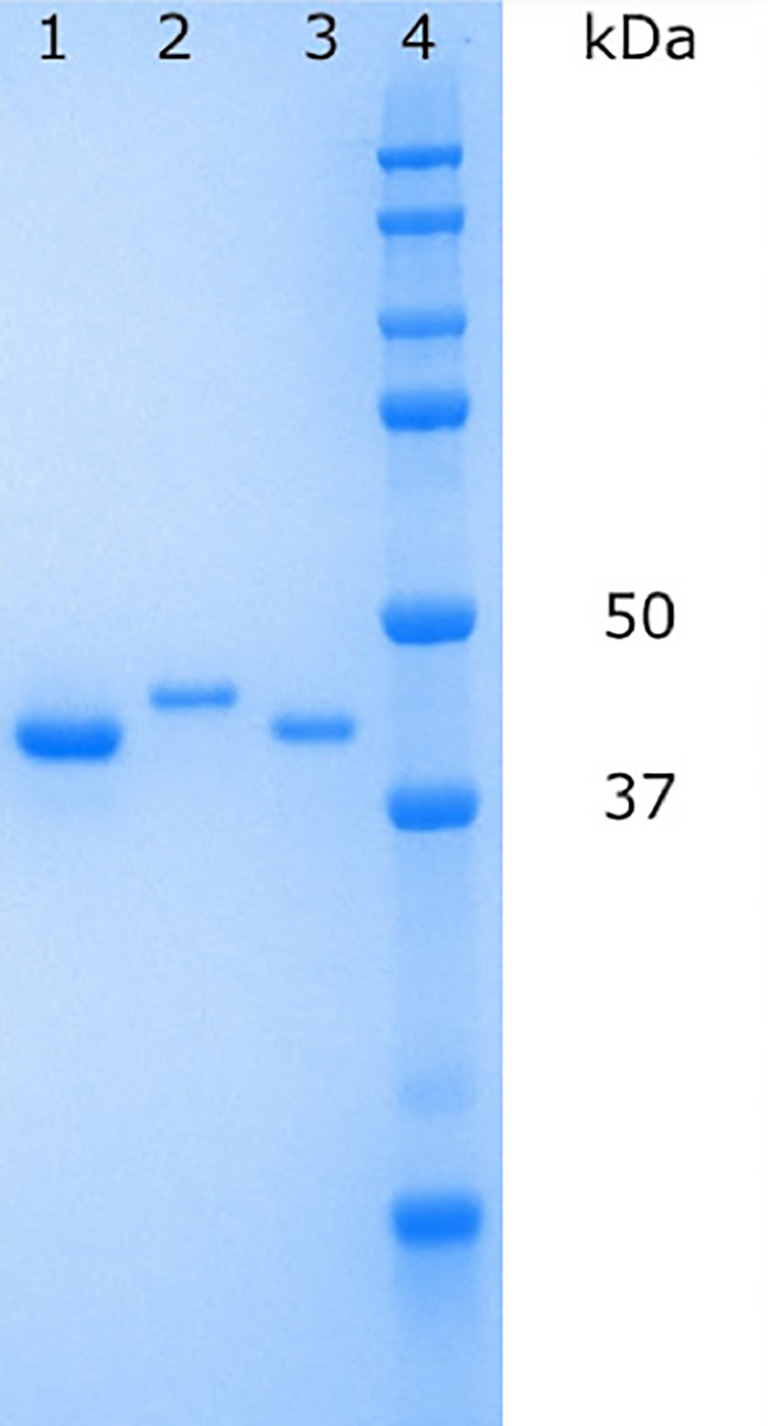
Protein samples on an SDS−PAGE gel. Lane 1: *Pa*FabB C164A (without His-tag) after inverse affinity chromatography, lane 2: 6-His-tagged *Pa*FabB C164A after SEC purification, lane 3: *Pa*FabB C164A (without His-tag) after SEC purification, lane 4: protein ladder.

His-tagged *Pa*FabB C161A was purified using affinity chromatography with a Ni column followed by size exclusion chromatography (SEC). To obtain FabB with a cleaved His-tag, the protein obtained after affinity chromatography was cleaved with TEV protease. The cleaved protein was separated from the protease and the tag by inverse affinity chromatography followed by SEC. In both cases, pure protein was obtained as judged by SDS-PAGE gel electrophoresis ( Figure 2). Typical yields for His-tagged *Pa*FabB C161A were 26 mg/L and for cleaved *Pa*FabB C161A 7 mg/L.

### Crystallization of *Pa*FabB C161A

**Figure 3.**
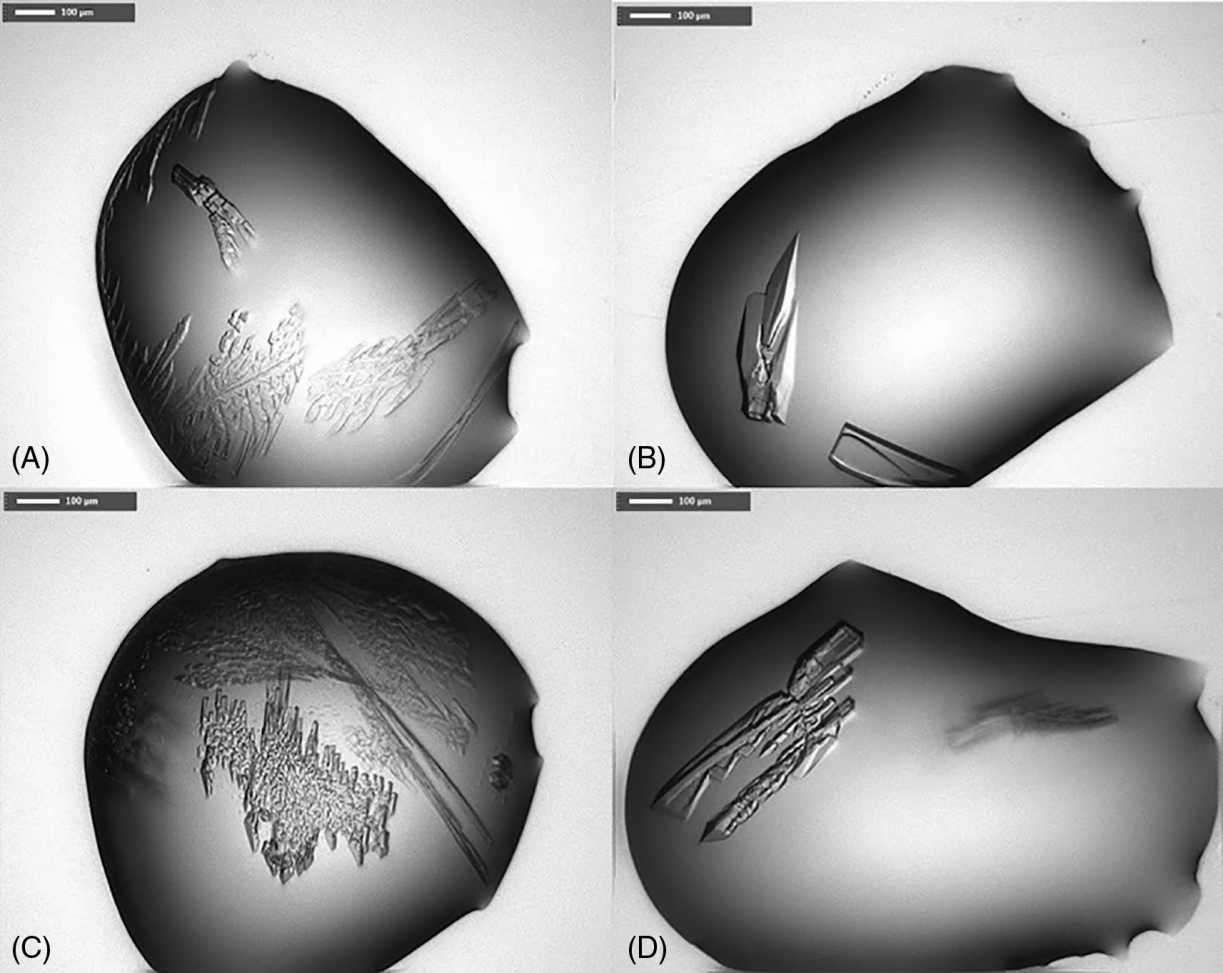
Selected crystals obtained from various screens. A) condition F3 from LFS, B) G3 from LFS, C) F3 from PACT premier, D) G3 from PACT premier (for composition of crystallization buffer see Table 1).

**Figure 4.**
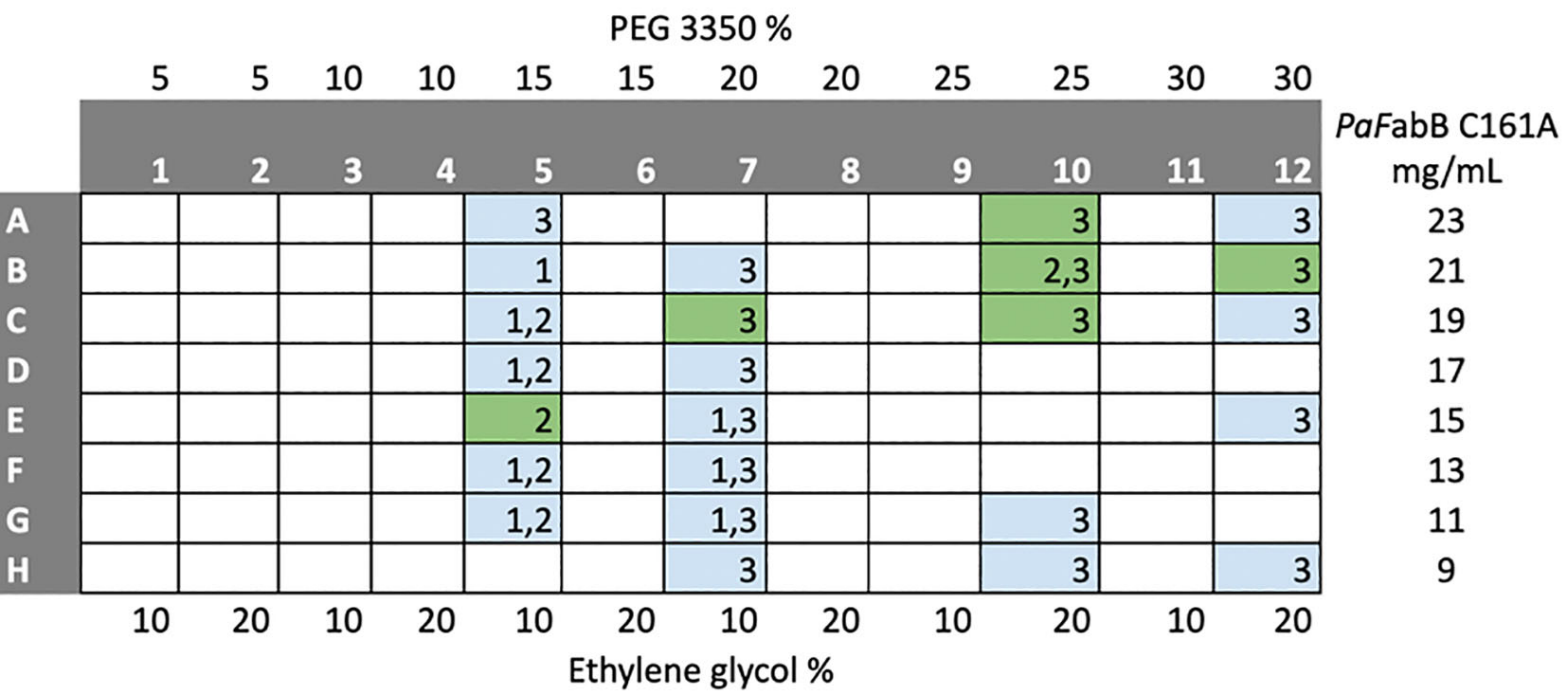
Plate layout for optimization of crystallization conditions. The numbers in the cells indicate the ratio between protein solution and crystallization buffer in the drops (drop 1-1:1 ratio, drop 2-1:2 ratio, drop 3-2:1 ratio). Coloured cells indicate conditions from which crystal were harvested and mounted for diffraction experiments. Green cells indicate conditions under which diffracting crystals were obtained.

Crystallization trials of His-tagged *Pa*FabB C161A and cleaved *Pa*FabB C164A were attempted using the JCSG+, PACT premier, HELIX (only His-tagged *Pa*FabB C161A) and LFS screens. No promising crystallization conditions for His-tagged *Pa*FabB C161A were found using these screens. In contrast, 11 different conditions resulted in crystals of His-tag cleaved *Pa*FabB C164A ( Table 1, Figure 2). All of these conditions contained PEG 3350 between 20 and 25% and a number of conditions contained ethylene glycol. Further, the majority of the conditions contained 0.1 M Bis-Tris propane, and 0.2 M sodium iodide. Therefore, these components were kept for further optimization trials. The pH of the initial conditions varied from 5.5 to 8.5. As crystals grown in a buffer of pH 7.5 were visually judged to be more regular (e. g. the crystal shown in Figure 3B), this pH was fixed during optimization. These considerations resulted in an optimization matrix where the concentration of PEG 3350 was varied between 5 and 30% and the protein concentration between 9 and 23 mg/mL. Ethylene glycol was added to all conditions at either 10 or 20% while 0.2 M sodium iodide and 0.1 M Bis-Tris propane were fixed ( Figure 4). Under 32 conditions, crystals were obtained. These were mounted and used for diffraction experiments.

**Table 1.** Conditions in which crystals of PaFabB C161A were formed. (PEG-polyethylene glycol; EG-ethylene glycol.)

Well screen	Buffer	Salt	Precipitant 1	Precipitant 2
F2 LFS	0.1 M Bis Tris Propane pH 6.5	0.2 M Sodium bromide	20 % *w/v* PEG 3350	10 % *v/v* EG
F3 LFS	0.1 M Bis Tris Propane pH 6.5	0.2 M Sodium iodide	20 % *w/v* PEG 3350	10 % *v/v* EG
F4 LFS	0.1 M Bis Tris Propane pH 6.5	0.2 M Potassium thiocyanate	20 % *w/v* PEG 3350	10 % *v/v* EG
G3 LFS	0.1 M Bis Tris Propane pH 7.5	0.2 M Sodium iodide	20 % *w/v* PEG 3350	10 % *v/v* EG
E3 PACT premier		0.2 M Sodium iodide	20 % *w/v* PEG 3350	
F2 PACT premier	0.1 M Bis-Tris propane pH 6.5	0.2 M Sodium bromide	20 % *w/v* PEG 3350	
F3 PACT premier	0.1 M Bis-Tris propane pH 6.5	0.2 M Sodium iodide	20 % *w/v* PEG 3350	
G2 PACT premier	0.1 M Bis-Tris propane pH 7.5	0.2 M Sodium bromide	20 % *w/v* PEG 3350	
G3 PACT premier	0.1 M Bis-Tris propane pH 7.5	0.2 M Sodium iodide	20 % *w/v* PEG 3350	
B2 JCSG+		0.2 M Sodium thiocyanate	20 % *w/v* PEG 3350	
D6 JCSG+	0.1 M Tris pH 8.5	0.2 M Magnesium chloride hexahydrate	20 % *w/v* PEG 8000	
H10 JCSG+	0.1 M BIS-Tris pH 5.5	0.2 M Ammonium acetate	25 % *w/v* PEG 3350	

Six different conditions led to well-diffracting crystals ( Figure 4). For these, data sets with resolutions between 2 and 1.3 Å could be collected. For the best diffracting crystal, the crystal structure was determined using a homology model created based on *Vibrio cholerae* FabB ( *Vc*FabB PDB Id 4XOX) as search model ( Table 2). The crystal was in the space group C 2 2 21 and contained 2 protein molecules in the asymmetric unit.

**Table 2.** Data-collection and refinement statistics of *Pa*FabB C161A. Values in parentheses are for the highest resolution shell.

**Data collection and processing**	
Space group	C 2 2 21
a, b, c (Å)	74.23, 102.30, 188.77
α, β, γ (°)	90.00 90.00 90.00
Solvent content (%)	40
**Diffraction data**	
Resolution range (Å)	47.2-1.3 (1.38-1.30)
Unique reflections	339190 (54366)
Multiplicity	6.84 (6.58)
R merge (%)	5.8 (49.4)
Completeness (%)	99.6 (98.6)
I/sigI	19.11 (3.45)
**Refinement**	
R work/R free	0.114/0.137
Quaternary structure	dimer
Protein residues (in a dimer)	808
Water molecules (in a dimer)	492
Ions (in a dimer)	Iodide (11), Chloride (6)
Ligands (in a dimer)	1,2-ETHANEDIOL (18)
**R.m.s.d.s**	
Bonds (Å)	0.013
Angles (Å)	1.75
**Ramachandran plot,residues in (%)**	
Favoured regions	790 (96.02%)
Allowed regions	33 (3.98%)
Outlier regions	0 (0%)
**Average B factors (Å ^2^)**	
Protein atoms	16.8
Ions, Ligands, Waters	30.7, 27.3, 30.0
**PDB code**	7PPS

### Crystal structure of *Pa*FabB C161A

**Figure 5.**
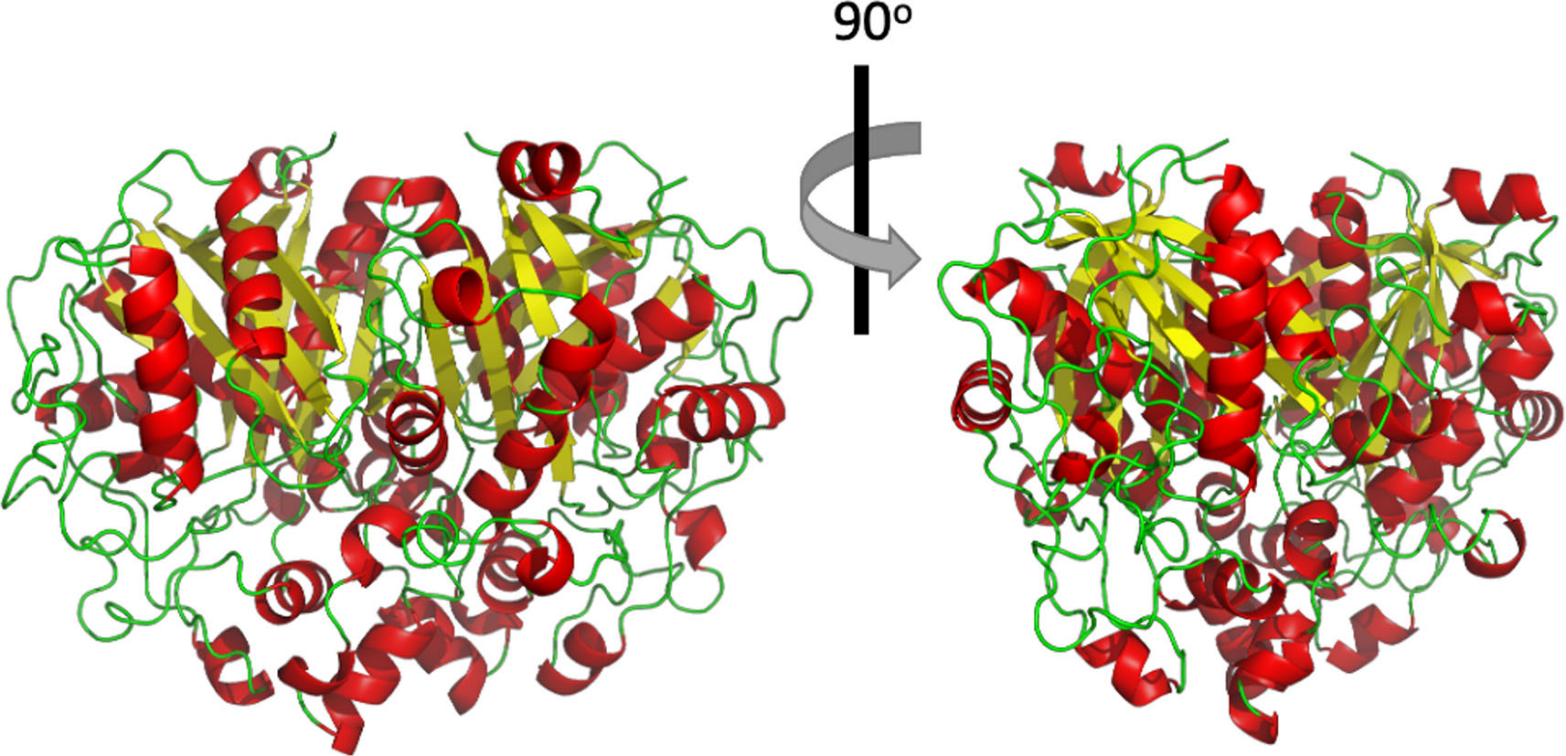
Cartoon representation showing the tertiary structure of *Pa*FabB C161A. Beta sheets and alpha helices are shown in yellow and red colour respectively while loops are shown in green.

**Figure 6.**
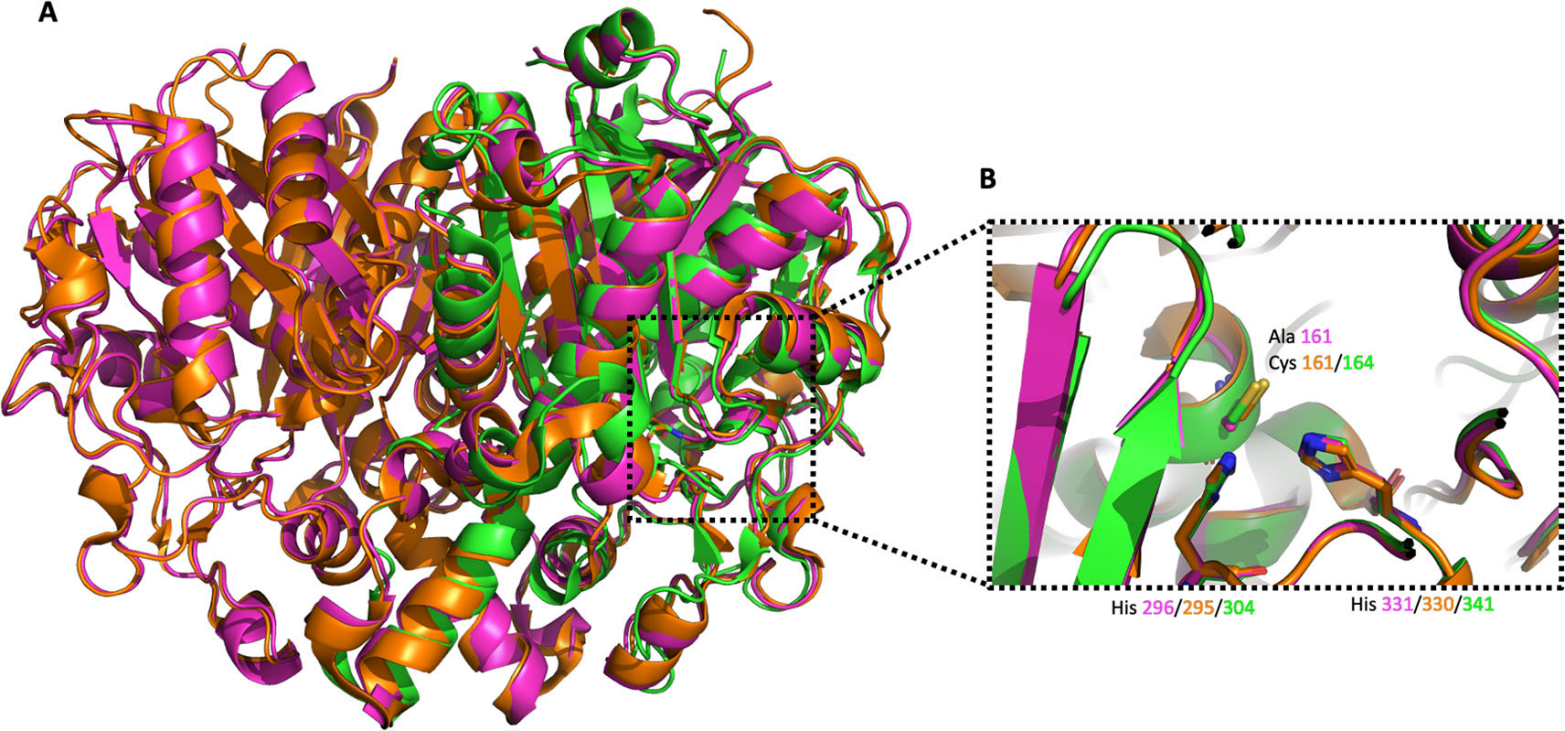
Alignment of *Vc*FabB (PDB Id 4XOX), *Pa*FabF (PDB Id 4JPF) and *Pa*FabB C161A. A) The three different enzymes are shown in orange, green and magenta cartoon style, respectively. B) Alignment of the active site catalytic triad of *Vc*FabB, w. t. *Pa*FabF and *Pa*FabB C161A.

*P*aFabB C161A has the same overall fold as observed before for FabB and FabF from other organisms ( Figure 5 and Figure 6A). The rmsd between *Pa*FabB C161A and *Vc*FabB (the protein with the highest sequence identity in the PDB (72%), PDB Id 4XOX) is 0.424 Å while the rmsd to the w.t. *Pa*FabF (sequence identity 41%, PDB Id 4JPF) is 0.843 Å. The two catalytic histidines, His 296 and His 331, are highly conserved and well aligned with the catalytic histidines from both *Vc*FabB as well as the ones from *Pa*FabF ( Figure 6B).

**Figure 7.**
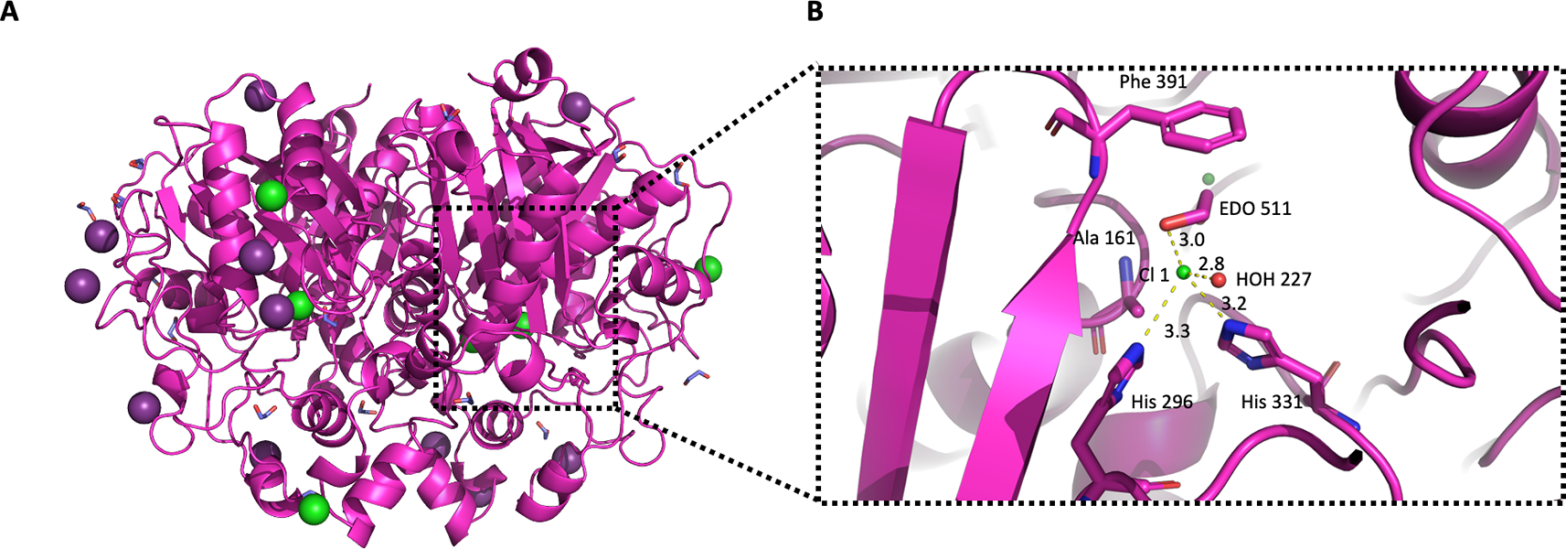
Crystal structure of *Pa*FabB C161A. A) The structure of *Pa*FabB is shown as magenta cartoon style. Iodine and chloride ions are shown with deep purple and green colour respectively. B) Active site residues are shown as magenta sticks, water molecules and chloride ions are shown as red and green spheres, respectively, while the distances between the chloride ion Cl 1 and the neighbouring molecules are shown as yellow dashed lines.

Due to the high concentration of ethylene glycol (20% *v/v*) and salt in the well and protein buffers, respectively (150 mM NaCl and 200 mM NaI), 18 ethylene glycol molecules and 11 ions (Cl ^-^ and I ^-^) were identified and placed in the crystal structure of *Pa*FabB C161A during refinement ( Figure 7A). Some of these molecules were found to bind in the active site of the protein ( Figure 7B). The chloride ion Cl 1 binds tightly (B factor for Cl 1 is 18 Å ^2^, average B-factor for protein atoms is 16.8 Å ^2^, average ions B factors is 30.7 Å ^2^) in the active site of chain B, in close proximity to the catalytic residues His 296 (3.3 Å) and His 331 (3. 2Å). Moreover, Cl 1 forms two additional interactions with an ethylene glycol (EDO511, average B factor 18 Å ^2^) and a water molecule (HOH227) in the active site.

### Active site and differences to *Pa*FabF

**Figure 8.**
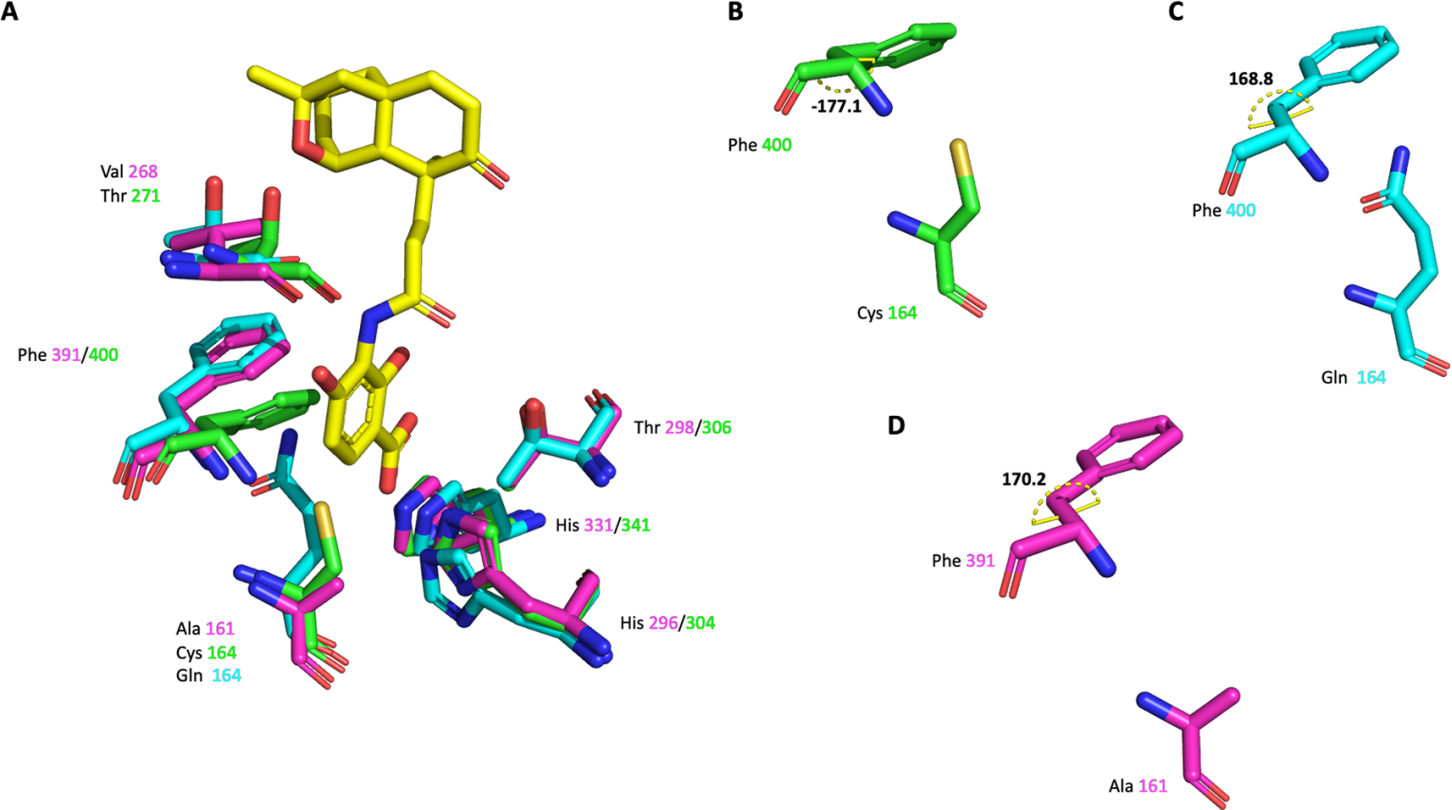
Alignment of *Pa*FabF (PDB Id 4JPF), *Pa*FabF C164Q (PDB Id 7OC1) and *Pa*FabB C161A. A) The active site residues of the three different enzymes are shown as green, cyan and magenta sticks, respectively. Platensimycin binding to *Pa*FabF C164Q (PDB Id 7OC1) is shown as yellow sticks. Side chain conformation and dihedral angle C-CA-CB-CG of Phe391/400 is shown in B) for *Pa*FabF C) for *Pa*FabF C164Q and D) for *Pa*FabB C161A.

**Figure 9.**
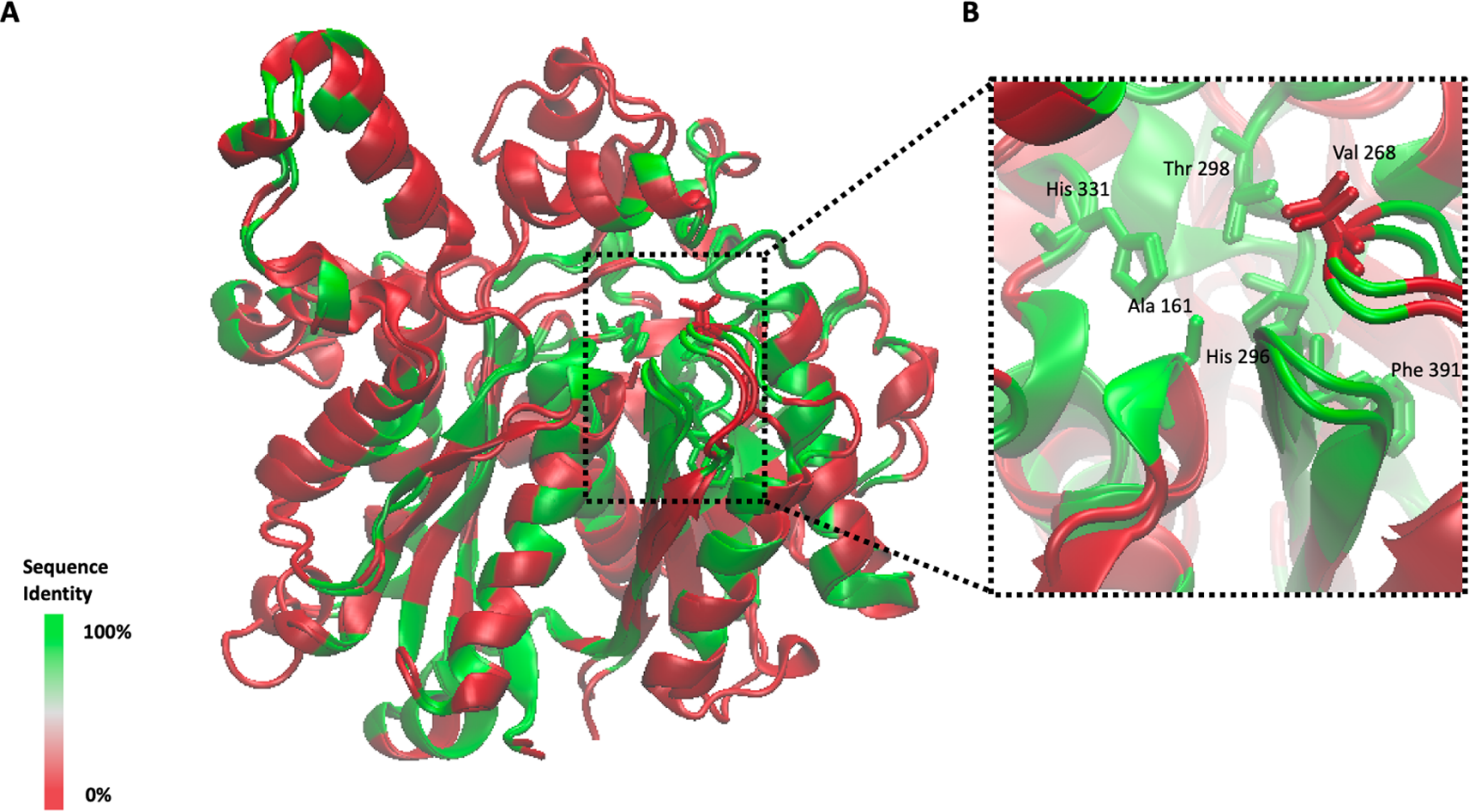
Sequence identity conservation between *Pa*FabB C161A and *Pa*FabF (PDB Id 4JPF). A) Alignment between chain A of *Pa*FabF and *Pa*FabB C161A is shown as cartoon style and color-coded based on the sequence identity between the two isoenzymes. B) Zoom in showing the active site and active site residues. Residue labels and numbering are based on *Pa*FabB only.

Phe 400/391 (numbering based on *Pa*FabF/ *Pa*FabB) was previously identified, as one of the highly conserved active site residues, to play a pivotal role in substrate specificity and ligand binding, by adopting a different conformation between the w.t. and the intermediate state of the FabF enzyme. ^9^^,^^12^ In w. t. *Pa*FabF (PDB Id 4JPF, Figure 8A and B), Phe400 is in a ‘closed’ conformation (dihedral angle C-CA-CB-CG = -177.1 ^o^). When mutating the catalytic residue Cys 164 to Gln 164 (PDB Id 7OC1 – Figure 8A and C) the enzyme has been shown to mimic the intermediate state and to trap the Phe 391 into the ‘open’ conformation (dihedral angle C-CA-CB-CG = 168.8 ^o^). Here, the catalytic residue Cys 161 of *Pa*FabB was mutated to Ala 161. As can be seen from the crystal structure ( Figure 8A and D), Phe 391 adopts the ‘open’ conformation as expected for an intermediate-mimicking FabB variant (dihedral angle C-CA-CB-CG = 170.2 ^o^). Although, the overall sequence identity between *Pa*FabB and *Pa*FabF is only 41%, the conservation in the active site is much higher ( Figure 9). Apart from Thr 271 in FabF that is mutated to Val 268 in FabB, all active site residues involved in hydrogen-bond interactions with platensimycin are conserved between the two enzymes ( Figure 8A). That makes it highly likely that ligands binding into the active site of FabF may also bind to FabB with a similar affinity, and thus opens up the possibility for the designing of dual inhibitors for both FabF and FabB that will lead to a complete inhibition of the last step of the fatty acid elongation cycle.

## Discussion

In this study, the first high-resolution crystal structure of *Pa*FabB C161A is reported. This structure can now serve as a template for the structure-based design of FabB inhibitors. The C161A mutation of FabB in this crystal structure causes Phe 391 to be in the ‘open’ conformation ( Figure 8) and allows targeting of the intermediate-acylated state of FabB; in a similar manner to the natural antibiotic platensimycin. Furthermore, due to the high conservation of the overall fold and the high sequence identity in the active site between the structure reported here with *Pa*FabF C164Q (PDB Id 7OC1), both structures can be used as a template for the design of novel dual FabF/B inhibitors.

## Methods

### Recombinant protein production and purification

The gene coding for *P. aeruginosa* PA14 FabB (ORF number (open reading frame): PA14_43690), with a single point mutation C161A was synthesised and cloned in a bacterial plasmid pET-28a(+)-TEV vector using the cloning sites NdeI/BamHI by Genscript. The plasmid had a DNA sequence coding for a 6-His-tag followed by a TEV cleavage site before *Pa*FabB. Seven different *E. coli* strains (OverExpress C41(DE3) SOLOs and C43(DE3) SOLOs from Biosearch technologies; BL-21(DE3), BL-21(DE3) pLysS, C41(DE3) pLysS and C43(DE3) pLysS from Lucigen, and Rosetta (DE3) pLysS from Merck) were heat-shock transformed with the synthesised plasmid. Expression of *Pa*FabB in each transformed cell line was tested as per manufacturer protocol.

*E.coli* Rosetta (DE3) pLysS competent cells yielded the highest protein expression, based on SDS-PAGE analysis, and were used as an expression system for large-scale protein production and purification. Transformed cells were inoculated in 50 mL of LB medium supplemented with kanamycin (30 μg/mL) and chloramphenicol (50 μg/mL) overnight at 310 K. Pre-culture stocks were prepared by mixing the overnight culture with glycerol (final concentration 40% *v/v*), aliquoted and kept in –80 °C until use. For large-scale expression, 0.1 mL of pre-culture stock was inoculated in 100 mL of LB medium supplemented with kanamycin (30 μg/mL) and chloramphenicol (50 μg/mL) overnight at 310 K. The entire volume was then transferred into 900 mL of LB-medium containing antibiotics and the cell growth continued until OD _600_ reached 0.7. Protein expression was then induced by adding IPTG to a final concentration of 1 mM and the expression continued for another 3-3.5 hours.

Cells were harvested by centrifugation (15 minutes, 5000 *g*, 277 K), resuspended in lysis buffer (20 mM Tris-HCl, 500 mM NaCl, 20 mM imidazole, 1 mM DTT, 10% glycerol ( *v/v*), pH 7.4) with addition of one tablet of Complete EDTA-free protease inhibitor cocktail (Roche) and incubated with magnet stirring for 60 minutes at 277 K. 20 U (units) of DNAse I (Sigma Aldrich) was added per cell pellet, before the mixture was sonicated on ice by an ultrasonic processor (Sonics, Vibra-Cell VC130) for a total of two minutes with 10 seconds pulses with amplitude 70. The debris and insoluble protein were pelleted by centrifugation at 15000 rpm, 277 K, for 30 minutes. The supernatant was collected and filtered with Whatman filter units 0.2 μM (GE healthcare) using a syringe. The protein was then purified using a Ni ^2+^ Sepharose High Performance HisTrap HP 5 mL column (GE Healthcare) with an increasing imidazole gradient from 0 to 500 mM. The fractions containing *Pa*FabB C161A were pooled and TEV protease was added to remove the affinity tag. The mixture was dialyzed with buffer (25 mM Tris–HCl pH 7.5, 150 mM NaCl) overnight at 277 K and the cleaved protein was purified by passage through a Ni ^2+^ HisTrap column. SEC was then performed on a HiLoad 26/600 Superdex 75 pg column (Cytiva) with equilibration buffer (20 mM Tris-HCl, 150 mM NaCl, 1 mM DTT, pH 7.4). Purity was confirmed by SDS–PAGE (Mini-PROTEAN TGX Stain-Free Precast Gel; Bio-Rad) and the final concentration of *Pa*FabB C161A was determined using a NanoDrop ND-1000 (Thermo Fisher Scientific).

### Crystallization and X-ray data collection

For crystallization trials JCSG+ (MD1-37), PACT premier (MD1-29) and LFS (Ligand Friendly Screen, MD1-122) crystallization screens from Molecular Dimensions were used. His-tag cleaved *Pa*FabB C161A (23 mg/mL) in 20 mM Tris-HCl, 150 mM NaCl, 1 mM DTT, pH 7.4, was mixed with well buffer in different ratios (2:1, 1:1 and 1:2) on a Triple Sitting Drop 96-well plate (TTP Labtech) using a crystallography Mosquito LCP (TTP LabTech). The plates were incubated at 20°C. Optimization ( Figure 4) of the initial hit conditions ( Table 2) was achieved by varying the precipitants and protein concentrations while keeping the salt and buffer concentration constant. Optimisation led to rod-shaped crystals (250 × 100 × 10 μm) in multiple drops ( Figure 3).

Crystals with a final concentration of precipitant lower than 25%( *w/v*) were cryoprotected with a mixture consisting of the crystallization buffer and Cryomix 9 from CryoSol MD1-90 (Molecular Dimensions) (final composition of the cryomixture: 0.2 M NaI, 0.1 M Bis-Tris propane pH 7.5, 5%( *w*/ *v*) PEG 3350, 10% ( *v*/ *v*) EG 5 % ( *v/v*), diethylene glycol, 5 % ( *v/v*) 1,2-propanediol, 5 % ( *v/v*) dimethyl sulfoxide, 5 % ( *v/v*) glycerol, 5 mM NDSB 201 (3-(1-Pyridinio)-1-propanesulfonate), 5 % ( *v/v*) 1,4-dioxane) prior to flash-cooling in liquid nitrogen.

X-ray data were collected from single crystals at the DESY synchrotron (Hamburg, Germany) at the P11 high-throughput MX beamline. In each case, crystals were maintained at 100 K and the X-ray wavelength was 0.976246 Å. Data were processed with the automatic data processing pipeline of P11 beamline, using XDS. ^13^

### Structure solution and refinement

The structure was solved by molecular replacement using Dimple ^14^ from the CCP4i2 suite. ^15^ As search model, a homology model generated from wt. *Vc*FabB (PDB Id 4XOX) with 72% sequence identity was used. Refinement was performed using REFMAC5 ^16^ while inspection of electron-density and difference density maps and model manipulation was achieved using *Coot.* ^17^ During refinement, water molecules, ions and side-chain conformers were included. The model geometry was assessed using *MolProbity,* ^18^ the PDB redo server ^19^ and the PDB validation tools. The crystallographic data and refinement statistics are listed in Table 2. The figures were generated with *PyMOL* v.2.4.1 (Schrödinger, LLC) and VMD v.1.9.3. ^20^

## Data availability

Protein Data Bank: The crystal structure of *Pa*FabB C161A with the PDB Id 7PPS, https://doi.org/10.2210/pdb7PPS/pdb. ^21^
