# Crystal structure of *Pseudomonas aeruginosa* FabB C161A, a template for structure-based design for new antibiotics

**DOI:** 10.12688/f1000research.74018.2

**Published:** 2022-01-10

**Authors:** Vladyslav Yadrykhins'ky, Charis Georgiou, Ruth Brenk

**Affiliations:** 1 Department of Biomedicine, University of Bergen, Bergen, 5020, Norway

**Keywords:** crystal structure, 3-oxoacyl-[acyl-carrier-protein] synthase 1, FabB, antibiotics

## Abstract

**Background**: FabB (3-oxoacyl-[acyl-carrier-protein] synthase 1) is part of the fatty acid synthesis II pathway found in bacteria and a potential target for antibiotics. The enzyme catalyses the Claisen condensation of malonyl-ACP (acyl carrier protein) with acyl-ACP via an acyl-enzyme intermediate. Here, we report the crystal structure of the intermediate-mimicking *Pseudomonas aeruginosa *FabB ( *Pa*FabB) C161A variant.

**Methods**: His-tagged *Pa*FabB C161A was expressed in *E. coli *Rosetta DE3 pLysS cells, cleaved by TEV protease and purified using affinity and size exclusion chromatography. Commercial screens were used to identify suitable crystallization conditions which were subsequently improved to obtain well diffracting crystals.

**Results**: We developed a robust and efficient system for recombinant expression of *Pa*FabB C161A. Conditions to obtain well diffracting crystals were established. The crystal structure of *Pa*FabB C161A was solved by molecular replacement at 1.3 Å resolution. Binding site comparison between *Pa*FabB and *Pa*FabF revealed a conserved malonyl binding site but differences in the fatty acid binding channel.

**Conclusions**: The *Pa*FabB C161A crystal structure can be used as a template to facilitate the design of FabB inhibitors.

## Introduction

New antibiotics are urgently needed to maintain the high standard of living that we have got accustomed to as the antibiotics of today are losing effectiveness faster than they are being replaced by new treatment options. ^1^

**Figure 1.**
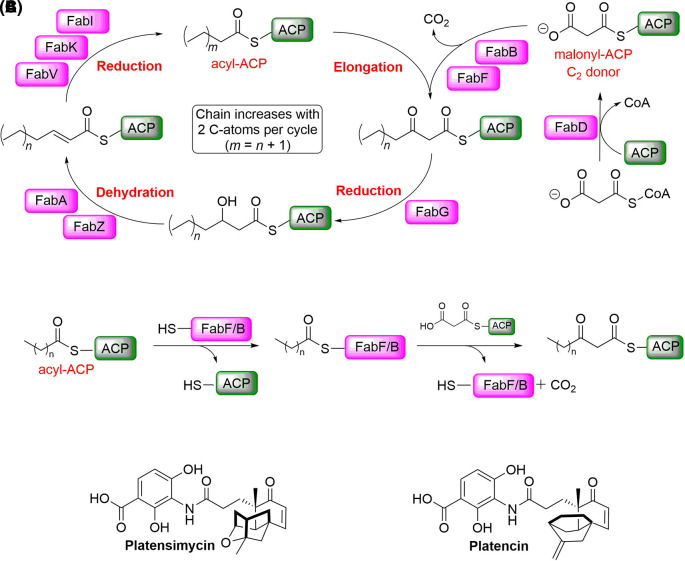
FAS II pathway and its inhibitors. A) Schematic overview of the elongation part of the FAS II pathway. B) Condensation reaction catalysed by FabF/B. (ACP: acyl carrier protein). C) Platensimycin and platencin have been reported as dual FabF/B inhibitors.

If no action is taken, by 2050 infections caused by drug-resistant pathogens will kill 10 million people a year worldwide, more than currently die from cancer. ^2^ A possible source for new targets for antibiotics is the fatty acid synthesis (FAS II) pathway ( Figure 1A). ^3^ In this pathway, fatty acid synthesis is carried out by a series of monofunctional enzymes which are highly conserved among microbial pathogens. Genes coding for enzymes in the FAS II pathway have been found to be essential for *P. aeruginosa* in several genetic screens, including the gene for FabB (3-oxoacyl-[acyl-carrier-protein] synthase 1). ^4^^–^^8^

**Figure 2.**
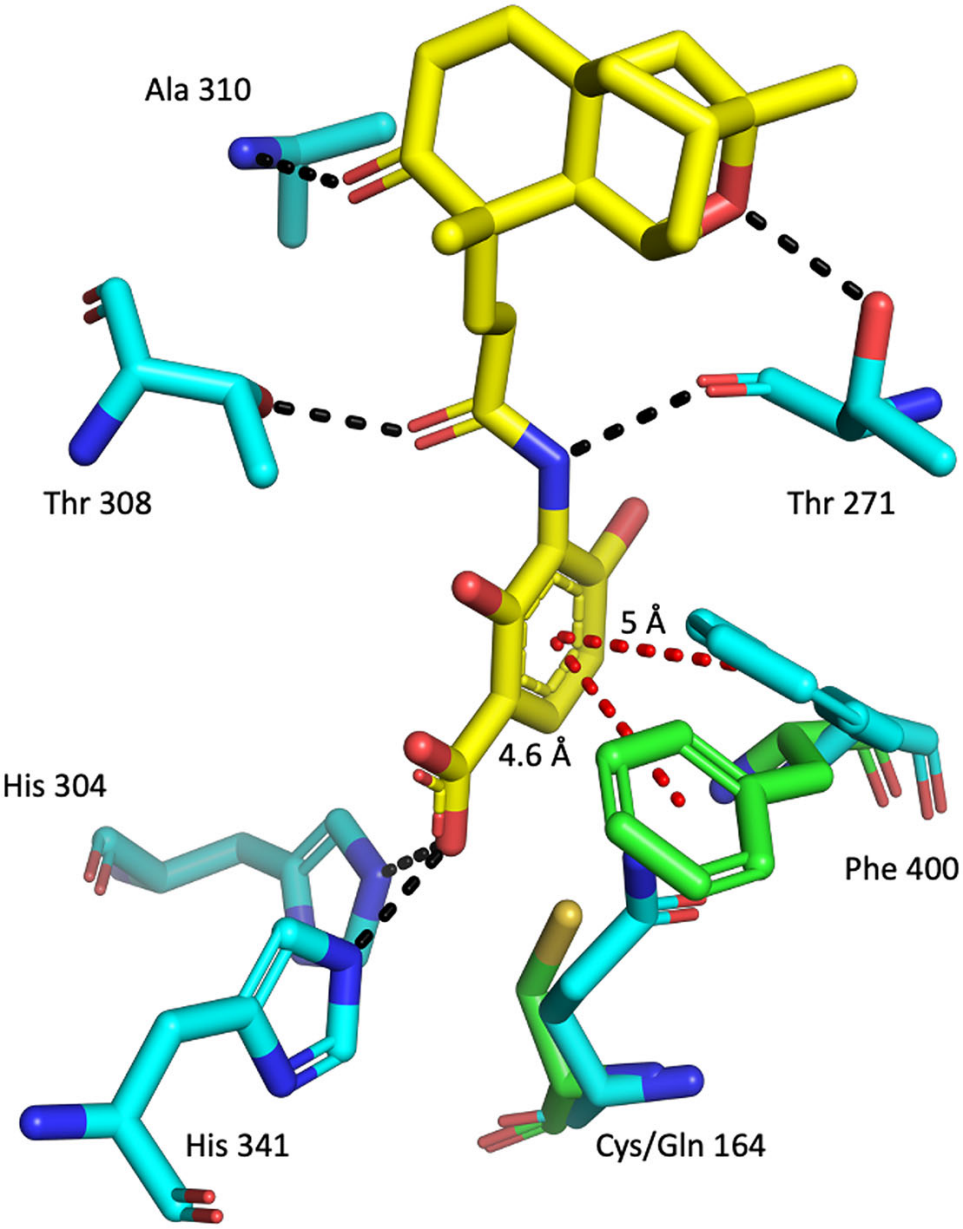
The malonyl binding site of FabF. Alignment of apo w. t. *Pa*FabF (green sticks – PDB ID: 4JPF , for clarity only Phe400 and Cys164 is shown) and *Pa*FabF C164Q (cyan sticks – PDB ID: 7OC1) in complex with platensimycin (yellow sticks). Hydrogen bonds are indicated as black dashed lines and aromatic interactions as red dashed lines. Compared to the apo structure, Phe400 is rotated in the holo structure to create space for the ligand to bind.

Both, FabB and FabF (3-oxoacyl-[acyl-carrier-protein] synthase 2) catalyse the Claisen condensation of malonyl-ACP (acyl carrier protein) with acyl-ACP ( Figure 1B), but differ in substrate specificity for the fatty acid chain. ^3^ Platensimycin and platencin ( Figure 1C) have been reported as FabF and FabB inhibitors binding into the malonyl binding site. ^9^^,^^10^ However, it has been shown that these compounds do not bind potently to the apo-enzyme, but only to the lauryl-FabF/B intermediate ( Figure 1B) and to intermediate-mimicking variants. In these variants, the active site Cys is replaced with either Gln or Ala ( Figure 2). ^9^^,^^11^ In the Ala variant, the presumably negatively charged Cys in the w. t. form is replaced with a neutral residue, thus mimicking more closely the charge of the lauryl intermediate. In the Gln variant, the amide group in the side chain in addition mimics the acyl group of the intermediate ( Figure 1B). Both variants have been used to study binding of malonyl-competitive inhibitors to FabF.

To facilitate structure-based design of FAS II inhibitors, knowledge of the structures in this pathway is essential. Recently, we have reported the crystal structure of *Pa*FabF and the reaction intermediate-mimicking variant *Pa*FabF C164Q. ^12^ Here, we report the crystal structure of an intermediate-mimicking *Pa*FabB variant at 1.3 Å resolution. As in our hands *Pa*FabF C164A was more stable than *Pa*FabF C164Q and thus better suited for biophysical studies, we focused our efforts on *Pa*FabB C161A. ^13^

## Results and discussion

### Protein expression and purification

The gene coding for *P. aeruginosa* PA14 FabB C161A was synthesised and cloned in a bacterial plasmid pET-28a(+)-TEV vector after a DNA sequence coding for a 6-His-tag followed by a TEV protease cleavage site. To find good expression conditions, seven widely used *E. coli* strains were transformed with the plasmid (BL21 (DE3), BL21 (DE3) pLysS, C41 (DE3), C41 (DE3) pLysS, C43 (DE3), C43 (DE3) pLysS and Rosetta (DE3) pLysS) and screened for protein expression. The best results were obtained with Rosetta (DE3) pLysS cells (data not shown). Therefore, this cell line was used for all subsequent protein expression experiments.

**Figure 3.**
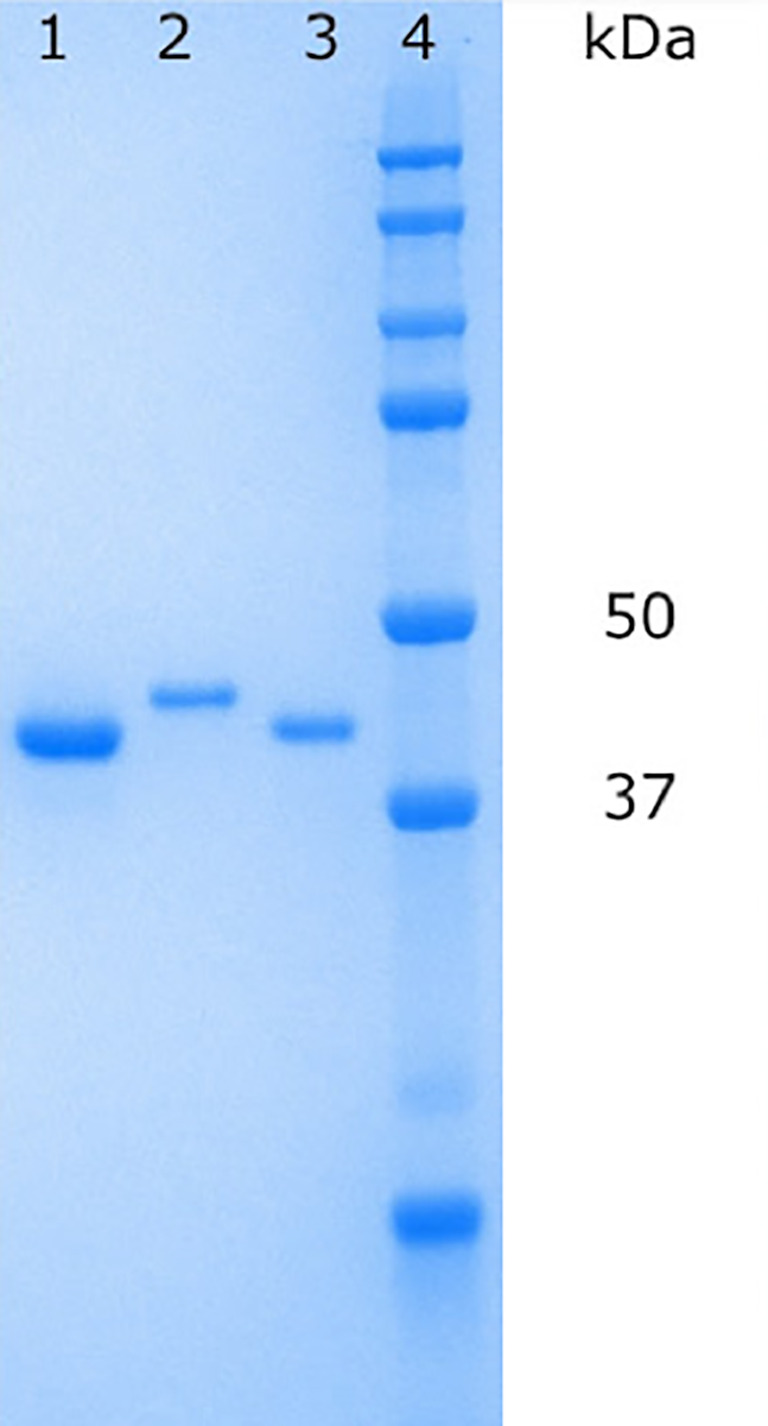
Protein samples on an SDS−PAGE gel. Lane 1: *Pa*FabB C161A (without His-tag) after inverse affinity chromatography, lane 2: 6-His-tagged *Pa*FabB C161A after SEC purification, lane 3: *Pa*FabB C161A (without His-tag) after SEC purification, lane 4: protein ladder.

His-tagged *Pa*FabB C161A was purified using affinity chromatography with a Ni column followed by size exclusion chromatography (SEC). To obtain FabB lacking the His-tag, the protein obtained after affinity chromatography was cleaved with TEV protease. The cleaved protein was separated from the protease and the tag by inverse affinity chromatography followed by SEC. In both cases, pure protein was obtained as judged by SDS-PAGE gel electrophoresis ( Figure 3). Typical yields for His-tagged *Pa*FabB C161A were 26 mg/L and for cleaved *Pa*FabB C161A 7 mg/L.

### Crystallization of *Pa*FabB C161A

**Figure 4.**
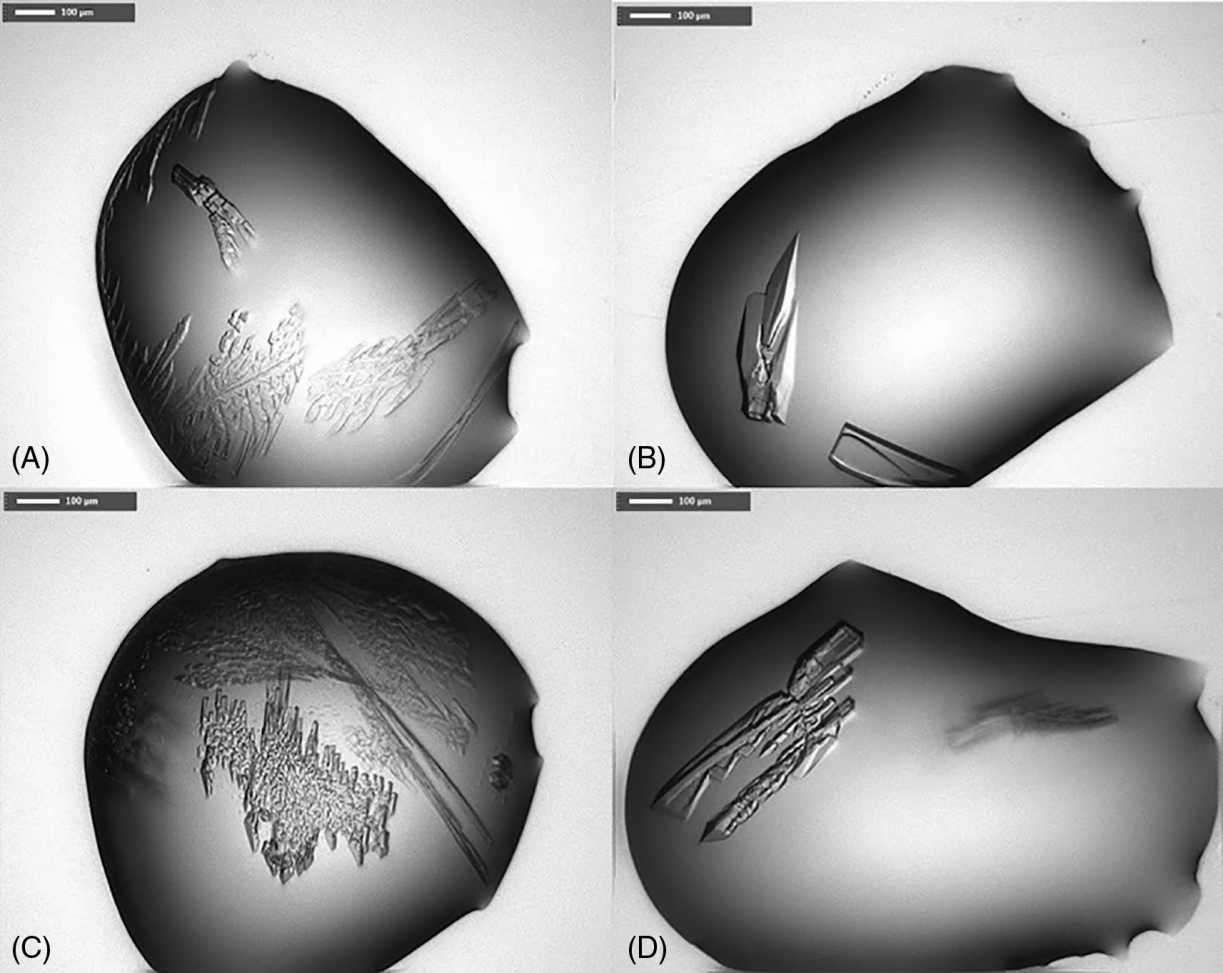
Selected *Pa*FabB C161A crystals obtained from various screens. A) condition F3 from LFS, B) G3 from LFS, C) F3 from PACT premier, D) G3 from PACT premier (for composition of crystallization buffer see Table 1).

**Figure 5.**
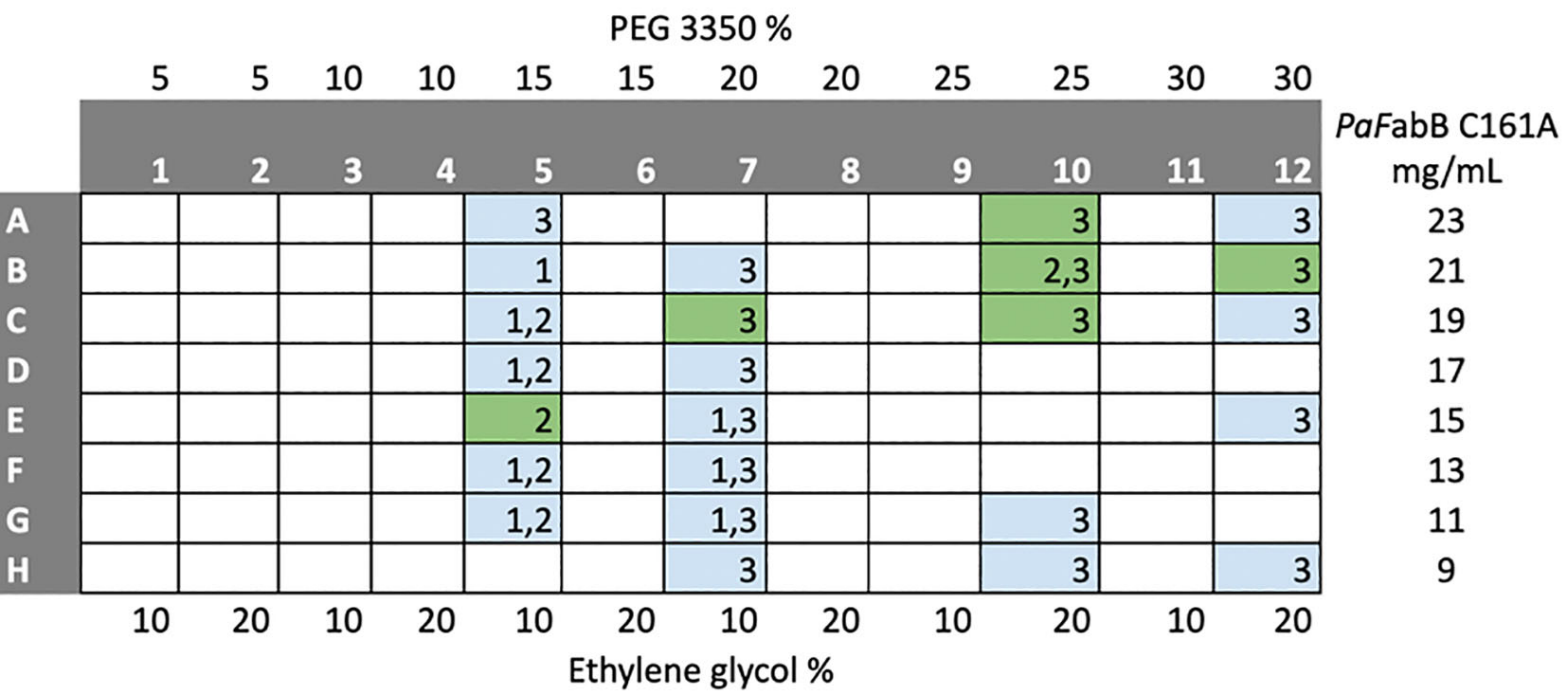
Plate layout for optimization of crystallization conditions. The numbers in the cells indicate the ratio between protein solution and crystallization buffer in the drops (drop 1-1:1 ratio, drop 2-1:2 ratio, drop 3-2:1 ratio). Coloured cells indicate conditions from which crystal were harvested and mounted for diffraction experiments. Green cells indicate conditions under which diffracting crystals were obtained.

Crystallization trials of His-tagged *Pa*FabB C161A and FabB C161A lacking the His-tag were attempted using the JCSG+, PACT premier, HELIX (only His-tagged *Pa*FabB C161A) and LFS screens. No promising crystallization conditions for His-tagged *Pa*FabB C161A were found using these screens. In contrast, 11 different conditions resulted in crystals of *Pa*FabB C161A lacking the His-tag ( Table 1, Figure 4). All of these conditions contained PEG 3350 between 20 and 25% and a number of conditions contained ethylene glycol. Further, the majority of the conditions contained 0.1 M Bis-Tris propane, and 0.2 M sodium iodide. Therefore, these components were kept for further optimization trials. The pH of the initial conditions varied from 5.5 to 8.5. As crystals grown in a buffer of pH 7.5 were visually judged to be more regular (e. g. the crystal shown in Figure 4B), this pH was fixed during optimization. These considerations resulted in an optimization matrix where the concentration of PEG 3350 was varied between 5 and 30% and the protein concentration between 9 and 23 mg/mL. Ethylene glycol was added to all conditions at either 10 or 20% while 0.2 M sodium iodide and 0.1 M Bis-Tris propane were fixed ( Figure 5). Under 32 conditions, crystals were obtained. These were mounted and used for diffraction experiments.

**Table 1.** Conditions in which crystals of PaFabB C161A (without His-tag) were formed. (PEG-polyethylene glycol; EG-ethylene glycol.)

Well screen	Buffer	Salt	Precipitant 1	Precipitant 2
F2 LFS	0.1 M Bis Tris Propane pH 6.5	0.2 M Sodium bromide	20% *w/v* PEG 3350	10% *v/v* EG
F3 LFS	0.1 M Bis Tris Propane pH 6.5	0.2 M Sodium iodide	20% *w/v* PEG 3350	10% *v/v* EG
F4 LFS	0.1 M Bis Tris Propane pH 6.5	0.2 M Potassium thiocyanate	20% *w/v* PEG 3350	10% *v/v* EG
G3 LFS	0.1 M Bis Tris Propane pH 7.5	0.2 M Sodium iodide	20% *w/v* PEG 3350	10% *v/v* EG
E3 PACT premier		0.2 M Sodium iodide	20% *w/v* PEG 3350	
F2 PACT premier	0.1 M Bis-Tris propane pH 6.5	0.2 M Sodium bromide	20% *w/v* PEG 3350	
F3 PACT premier	0.1 M Bis-Tris propane pH 6.5	0.2 M Sodium iodide	20% *w/v* PEG 3350	
G2 PACT premier	0.1 M Bis-Tris propane pH 7.5	0.2 M Sodium bromide	20% *w/v* PEG 3350	
G3 PACT premier	0.1 M Bis-Tris propane pH 7.5	0.2 M Sodium iodide	20% *w/v* PEG 3350	
B2 JCSG+		0.2 M Sodium thiocyanate	20% *w/v* PEG 3350	
D6 JCSG+	0.1 M Tris pH 8.5	0.2 M Magnesium chloride hexahydrate	20% *w/v* PEG 8000	
H10 JCSG+	0.1 M BIS-Tris pH 5.5	0.2 M Ammonium acetate	25% *w/v* PEG 3350	

Six different conditions led to well-diffracting crystals ( Figure 5). For these, data sets with resolutions between 2 and 1.3 Å could be collected. For the best diffracting crystal, the resolution was set limited based on the distance of the detector from the crystal and no data were discarded. Based on the CC1/2 and I/sigI values ( Table 2) it is likely this FabB crystal diffracted to an even higher resolution than 1.3 Å. The crystal structure was determined using a homology model created based on *Vibrio cholerae* FabB ( *Vc*FabB, PDB Id 4XOX) as search model. The crystal was in the space group C 2 2 21 and contained 2 protein molecules in the asymmetric unit.

**Table 2.** Data-collection and refinement statistics of *Pa*FabB C161A. Values in parentheses are for the highest resolution shell.

**Data collection and processing**	
Space group	C 2 2 21
a, b, c (Å)	74.23, 102.30, 188.77
α, β, γ (°)	90.00 90.00 90.00
Solvent content (%)	40
**Diffraction data**	
Resolution range (Å)	47.2-1.3 (1.38-1.30)
Unique reflections	339190 (54366)
Multiplicity	13.4 (12.3)
R merge (%)	5.8 (49.4)
Completeness (%)	99.7 (97.3)
I/sigI	22.4 (4.6)
CC (1/2)	99.9 (92.8)
**Refinement**	
R work/R free	0.114/0.137
Quaternary structure	dimer
Protein residues (in a dimer)	808
Water molecules (in a dimer)	492
Ions (in a dimer)	Iodide (11), Chloride (6)
Ligands (in a dimer)	1,2-ETHANEDIOL (18)
**R.m.s.d.s**	
Bonds (Å)	0.013
Angles (Å)	1.75
**Ramachandran plot,residues in (%)**	
Favoured regions	790 (96%)
Allowed regions	33 (4%)
Outlier regions	0 (0%)
**Average B factors (Å ^2^)**	
Protein atoms	16.8
Ions, Ligands, Waters	30.7, 27.3, 30.0
**PDB code**	7PPS

### Crystal structure of *Pa*FabB C161A

**Figure 6.**
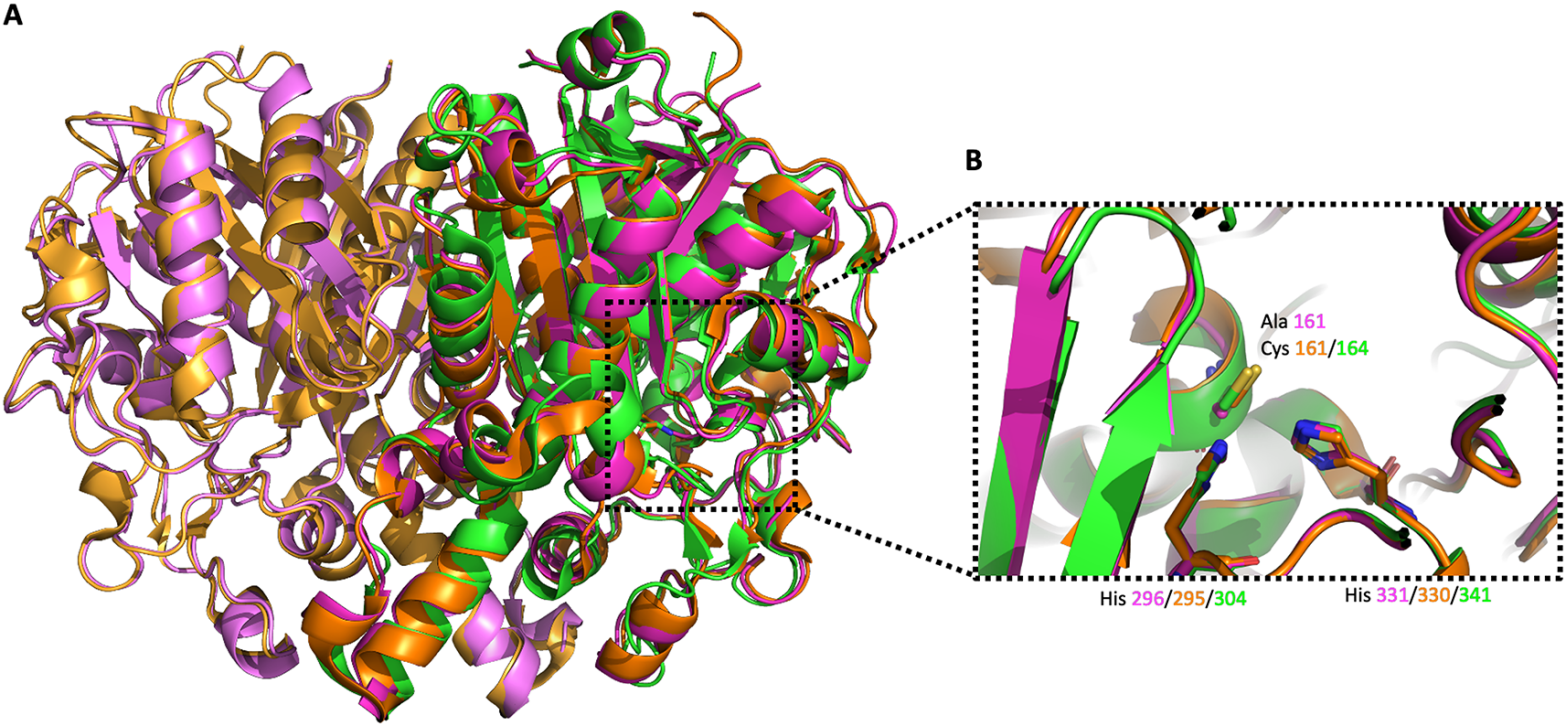
Alignment of *Vc*FabB (PDB Id 4XOX) dimer, *Pa*FabF (4JPF) monomer and *Pa*FabB C161A (7PPS) dimer. A) The three different enzymes are shown in orange/light orange, green and magenta/light magenta cartoon style, respectively. B) Alignment of the active site catalytic triad of *Vc*FabB, w. t. *Pa*FabF and *Pa*FabB C161A.

*P*aFabB C161A crystallized as a dimer and has the same overall fold as observed before for FabB and FabF from other organisms ( Figure 6). The rmsd between *Pa*FabB C161A and *Vc*FabB (the protein with the highest sequence identity in the PDB (72%), PDB Id 4XOX) is 0.42 Å while the rmsd to w. t. *Pa*FabF is 0.84 Å (sequence identity 41%, PDB Id 4JPF). The two catalytic histidines, His296 and His331, are highly conserved and well aligned with the catalytic histidines from both *Vc*FabB and *Pa*FabF ( Figure 6B).

**Figure 7.**
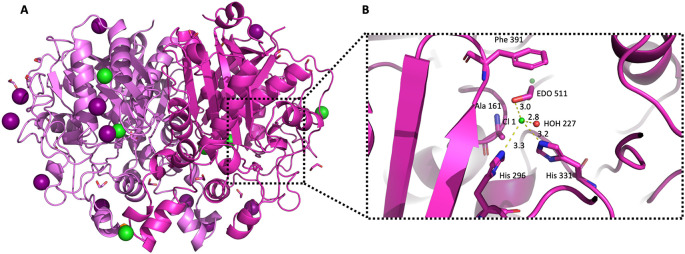
Crystal structure of *Pa*FabB C161A highlighting bound buffer components. A) The structure the of *Pa*FabB C161A homodimer is shown in carton style coloured in magenta/light magenta. Iodine and chloride ions are shown with deep purple and green colour, respectively. B) Active site residues are shown as magenta sticks, water molecules and chloride ions are shown as red and green spheres, respectively, while the distances between the chloride ion Cl1 and the neighbouring molecules are shown as yellow dashed lines.

Due to the high concentration of ethylene glycol (20% *v/v*) and salt in the well and protein buffers, respectively (150 mM NaCl and 200 mM NaI), 18 ethylene glycol molecules and 11 ions (Cl ^-^ and I ^-^) were identified and placed in the crystal structure of *Pa*FabB C161A during refinement ( Figure 7A). Some of these molecules were found to bind in the active site of the protein ( Figure 7B). The chloride ion Cl 1 binds tightly (B factor for Cl 1 is 18 Å ^2^, average B-factor for protein atoms is 16.8 Å ^2^, average ions B factors is 30.7 Å ^2^) in the active site of chain B, in close proximity to the catalytic residues His296 (3.3 Å) and His331 (3. 2Å). Moreover, Cl 1 forms two additional interactions with an ethylene glycol (EDO511, average B factor 18 Å ^2^) and a water molecule (HOH227) in the active site.

### Comparison of malonyl binding site in *Pa*FabB and *Pa*FabF

**Figure 8.**
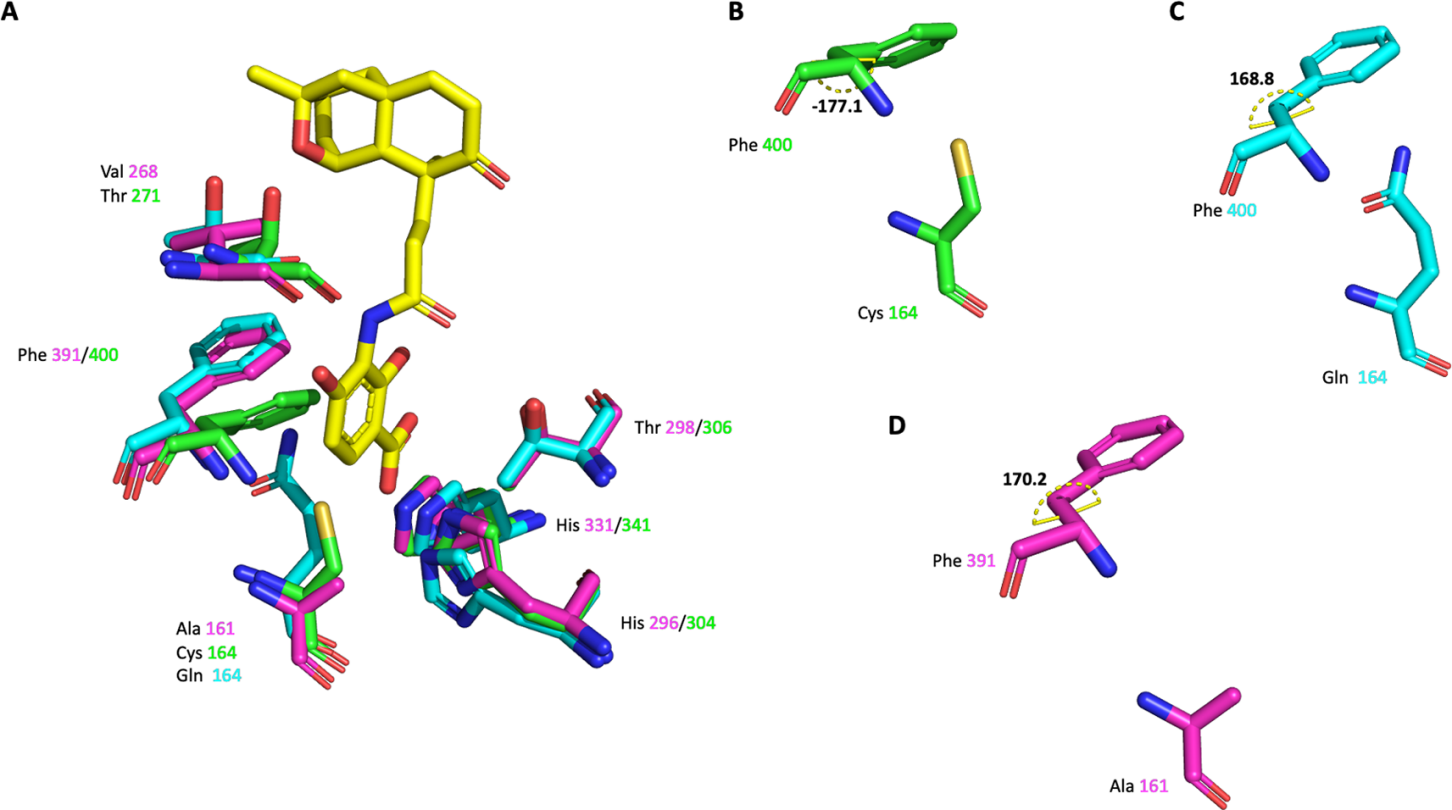
Alignment of the malonyl binding site of *Pa*FabF (PDB Id 4JPF), *Pa*FabF C164Q (7OC1) and *Pa*FabB C161A (7PPS). A) The active site residues of the three different enzymes are shown as sticks. Platensimycin binding to *Pa*FabF C164Q is shown as yellow sticks. Side chain conformation and dihedral angle C-CA-CB-CG of Phe391/400 is shown in B) for *Pa*FabF C) for *Pa*FabF C164Q and D) for *Pa*FabB C161A.

Although, the overall sequence identity between *Pa*FabB and *Pa*FabF is only 41%, the conservation in the malonyl binding site is much higher. Apart from Thr271 in FabF that is replaced by Val268 in FabB, all active site residues involved in hydrogen-bond interactions with platensimycin are conserved between the two enzymes ( Figure 8A). That makes it highly likely that ligands binding into this pocket in FabF may also bind to FabB with a similar affinity, and thus opens up the possibility for the designing of dual inhibitors for both FabF and FabB that will lead to a complete inhibition of the last step of the fatty acid elongation cycle.

The highly conserved Phe400/391 (numbering based on *Pa*FabF/ *Pa*FabB) in the malonyl binding site was previously identified to play a pivotal role in substrate specificity and ligand binding, as this residue adopts different conformations in the apo and the intermediate-binding state ( Figure 2). ^9^^,^^12^ In w. t. apo *Pa*FabF (PDB Id 4JPF, Figure 8A and B), Phe400 is in a ‘closed’ conformation (dihedral angle C-CA-CB-CG = -177.1 ^o^). Upon the mutation of the catalytic residue Cys164 to Gln (PDB Id 7OC1 – Figure 8A and C) or Ala, the enzyme has been shown to mimic the intermediate-binding state and to trap the Phe400 into the ‘open’ conformation (dihedral angle C-CA-CB-CG = 168.8 ^o^) as also found when a fatty acid is bound (e.g. PDB ID 2GFY). The reason for this is likely that the closed conformation is stabilized by a sulphur-pi interaction between the catalytic Cys and the Phe. ^14^ Once this interaction is disturbed through either binding the fatty acid or a point mutation of Cys, Phe adopts the then energetically more favourable open conformation. Here, the catalytic residue Cys161 of *Pa*FabB was mutated to Ala161. As can be seen from the crystal structure ( Figure 8A and D), Phe391 adopts the ‘open’ conformation as expected for an intermediate-mimicking FabB variant (dihedral angle C-CA-CB-CG = 170.2 ^o^).

### Comparison of the fatty acid chain binding channel in and in *Pa*FabB and *Pa*FabF

FabB and FabF catalyse the condensation of C4-C14 saturated fatty acids, but they show different levels of acceptance of unsaturated fatty acids. ^3^^,^^15^^,^^16^ FabB is able to catalyse the elongation of cis-3-decenoyl-ACP up to three times, and synthesise cis-5-dodecenoyl-ACP, cis-7-tetradodecenoyl-ACP and cis-9-hexadodecenoyl-ACP. Cells lacking FabB are auxotroph for unsaturated fatty acids, making FabB an essential gene for the bacteria. ^4^^,^^17^^,^^18^ In contrast, FabF but and not FabB was shown to be responsible for the condensation of cis-9-hexadodecenoyl-ACP to cis-11-octadodecenoyl-ACP, the last step for the synthesis of vaccenic acid. ^19^

Despite the fact that the crystal structures of both FabF and FabB have been published some time ago, it is still not clear what the molecular reasons for the observed fatty acid selectivity are. In a recent publication, a gating mechanism was proposed that regulates access to the fatty acid binding sites of FabB and FabF through a significant conformational change of two active site loops. However, based on the presented data no conclusions about the observed substrate selectivity can be drawn. ^20^

**Figure 9.**
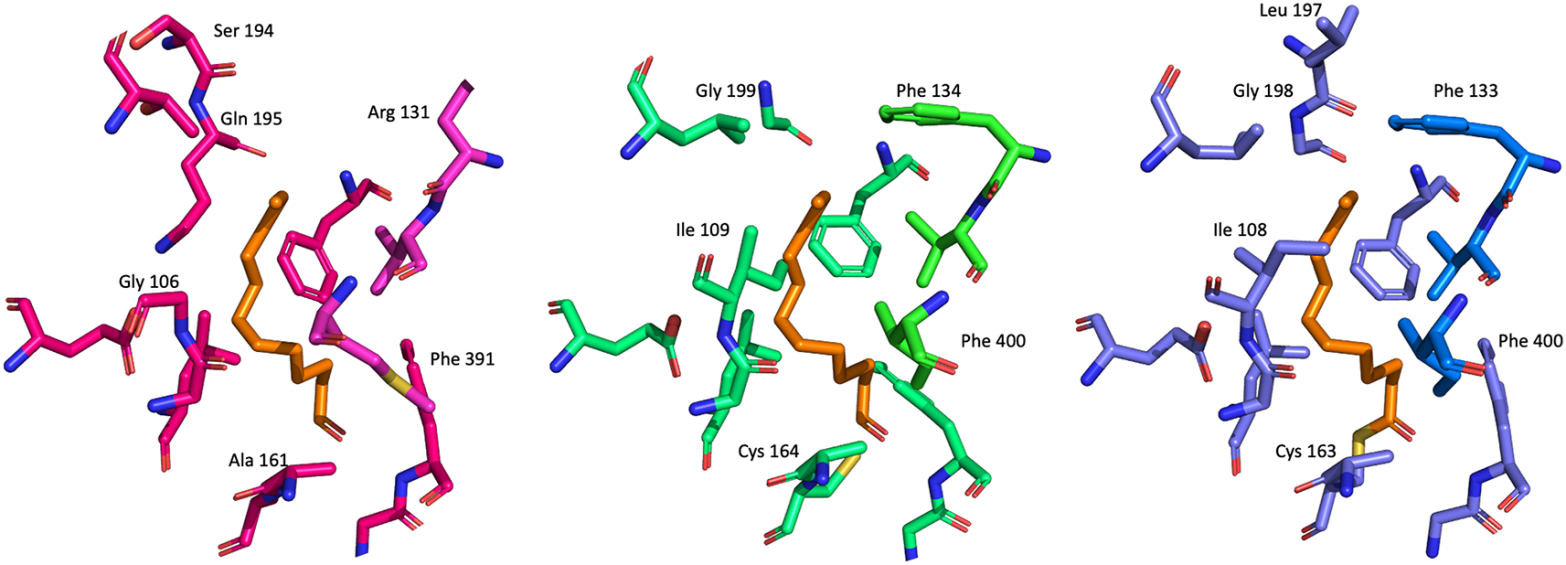
Comparison of the fatty acid binding channel residues of the homodimers of *Pa*FabF (PDB Id 4JPF), *Pa*FabB C161A (7PPS) and *Ec*FabF (2GFY). The fatty acid binding channel site residues of the three different enzymes are shown as blue/light blue, green/light green and magenta/light magenta sticks, respectively. C12 fatty acid binding to *Ec*FabF is superimposed with the remaining structures and shown as orange sticks.

The fatty acid binding channel is located at the interface of the homodimer ( Figure 9). No crystal structures of FabF/B with unsaturated acids for which the enzymes appear to be selective have been published so far. The entrance to the fatty acid channel is lined with identical residues in FabF and FabB. However, further into the channel, FabF has a Ile (108/109 *Ec*FabF/ *Pa*FabF numbering). This residue has been shown to rotate to allow the binding of fatty acids longer than C6. ^15^ The equivalent residues in *Pa*FabF is Gly106 making the channel wider in this part of the pocket and potentially also more flexible. This might explain the substrate promiscuity of FabB, but more detailed studies are needed to confirm this. A better understanding of the driving forces for the observed selectivity will also help with the design of selective of dual FabF/B inhibitors.

## Conclusions

In this study, the first high-resolution crystal structure of *Pa*FabB C161A is reported. This structure can now serve as a template for the structure-based design of FabB inhibitors. The C161A mutation of FabB in this crystal structure causes Phe 391 to be in the ‘open’ conformation ( Figure 8) and allows targeting of the intermediate-acylated state of FabB; in a similar manner to the natural antibiotic platensimycin. Furthermore, due to the high conservation of the overall fold and the high sequence identity in the malonyl binding site between the structure reported here with *Pa*FabF, the structures can be used as a template for the design of novel dual FabF/B inhibitors. In contrast, compounds extending deep into the fatty acid channel are likely to be selective, but more work is needed to get a better understanding of what drives the substrate selectivity in this channel.

## Methods

### Recombinant protein production and purification

The gene coding for *P. aeruginosa* PA14 FabB (ORF number (open reading frame): PA14_43690), with the single point mutation C161A was synthesised and cloned in a bacterial plasmid pET-28a(+)-TEV vector using the cloning sites NdeI/BamHI by Genscript. The plasmid had a DNA sequence coding for a 6-His-tag followed by a TEV protease cleavage site before *Pa*FabB. Seven different *E. coli* strains (OverExpress C41(DE3) SOLOs and C43(DE3) SOLOs from Biosearch technologies; BL-21(DE3), BL-21(DE3) pLysS, C41(DE3) pLysS and C43(DE3) pLysS from Lucigen, and Rosetta (DE3) pLysS from Merck) were heat-shock transformed with the synthesised plasmid. Expression of *Pa*FabB in each transformed cell line was tested as per manufacturer protocol.

*E. coli* Rosetta (DE3) pLysS competent cells yielded the highest protein expression, based on SDS-PAGE analysis, and were used as expression system for large-scale protein production and purification. Transformed cells were inoculated in 50 mL of LB medium supplemented with kanamycin (30 μg/mL) and chloramphenicol (50 μg/mL) overnight at 310 K. Pre-culture stocks were prepared by mixing the overnight culture with glycerol (final concentration 40% *v/v*), aliquoted and kept in –80 °C until use. For large-scale expression, 0.1 mL of pre-culture stock was inoculated in 100 mL of LB medium supplemented with kanamycin (30 μg/mL) and chloramphenicol (50 μg/mL) overnight at 310 K. The entire volume was then transferred into 900 mL of LB-medium containing antibiotics and the cell growth continued until OD _600_ reached 0.7. Protein expression was then induced by adding IPTG to a final concentration of 1 mM and the expression continued for another 3-3.5 hours.

Cells were harvested by centrifugation (15 minutes, 5000 *g*, 277 K), resuspended in lysis buffer (20 mM Tris-HCl, 500 mM NaCl, 20 mM imidazole, 1 mM DTT, 10% glycerol ( *v/v*), pH 7.4) with addition of one tablet of Complete EDTA-free protease inhibitor cocktail (Roche) and incubated with magnet stirring for 60 minutes at 277 K. 20 U (units) of DNAse I (Sigma Aldrich) was added per cell pellet, before the mixture was sonicated on ice by an ultrasonic processor (Sonics, Vibra-Cell VC130) for a total of two minutes with 10 seconds pulses with amplitude 70%. The debris and insoluble protein were pelleted by centrifugation at 15000 rpm, 277 K, for 30 minutes. The supernatant was collected and filtered with Whatman filter units 0.2 μM (GE healthcare) using a syringe. The protein was then purified using a Ni ^2+^ Sepharose High Performance HisTrap HP 5 mL column (GE Healthcare) with an increasing imidazole gradient from 0 to 500 mM. The fractions containing *Pa*FabB C161A were pooled and TEV protease was added to remove the affinity tag. The mixture was dialyzed with buffer (25 mM Tris–HCl pH 7.5, 150 mM NaCl) overnight at 277 K and the cleaved protein was purified by passage through a Ni ^2+^ HisTrap column. SEC was then performed on a HiLoad 26/600 Superdex 75 pg column (Cytiva) with equilibration buffer (20 mM Tris-HCl, 150 mM NaCl, 1 mM DTT, pH 7.4). Purity was confirmed by SDS–PAGE (Mini-PROTEAN TGX Stain-Free Precast Gel; Bio-Rad) and the final concentration of *Pa*FabB C161A was determined using a NanoDrop ND-1000 (Thermo Fisher Scientific). The extinction coefficient used was 0.666 (mg/mL) ^−1^ cm ^−1^ (calculated using the final protein sequence).

### Crystallization and X-ray data collection

For crystallization trials JCSG+ (MD1-37), PACT premier (MD1-29) and LFS (Ligand Friendly Screen, MD1-122) crystallization screens from Molecular Dimensions were used. *Pa*FabB C161A lacking the His-tag (23 mg/mL) in 20 mM Tris-HCl, 150 mM NaCl, 1 mM DTT, pH 7.4, was mixed with well buffer in different ratios (2:1, 1:1 and 1:2) on a Triple Sitting Drop 96-well plate (TTP Labtech) using a crystallography Mosquito LCP (TTP LabTech). The plates were incubated at 20°C. Optimization ( Figure 5) of the initial hit conditions ( Table 1) was achieved by varying the precipitants and protein concentrations while keeping the salt and buffer concentration constant. Optimisation led to rod-shaped crystals (250 × 100 × 10 μm) in multiple drops ( Figure 4).

Crystals with a final concentration of precipitant lower than 25% ( *w/v*) were cryoprotected with a mixture consisting of the crystallization buffer and Cryomix 9 from CryoSol MD1-90 (Molecular Dimensions) (final composition of the cryo-mixture: 0.2 M NaI, 0.1 M Bis-Tris propane pH 7.5, 5% ( *w*/ *v*) PEG 3350, 10% ( *v*/ *v*) EG 5% ( *v/v*), diethylene glycol, 5% ( *v/v*) 1,2-propanediol, 5% ( *v/v*) dimethyl sulfoxide, 5% ( *v/v*) glycerol, 5 mM NDSB 201 (3-(1-Pyridinio)-1-propanesulfonate), 5% ( *v/v*) 1,4-dioxane) prior to flash-cooling in liquid nitrogen.

X-ray data were collected from single crystals at the DESY synchrotron (Hamburg, Germany) at the P11 high-throughput MX beamline. In each case, crystals were maintained at 100 K and the X-ray wavelength was 0.976246 Å. Data were processed with the automatic data processing pipeline of P11 beamline, using XDS. ^21^

### Structure solution and refinement

The structure was solved by molecular replacement using Dimple ^22^ from the CCP4i2 suite. ^23^ As search model, a homology model generated from wt. *Vc*FabB (PDB Id 4XOX) with 72% sequence identity was used. Refinement was performed using REFMAC5 ^24^ while inspection of electron-density and difference density maps and model manipulation was achieved using *Coot.* ^25^ During refinement, water molecules, ions and side-chain conformers were included. The model geometry was assessed using *MolProbity,* ^26^ the PDB redo server ^27^ and the PDB validation tools. The crystallographic data and refinement statistics are listed in Table 2. The figures were generated with *PyMOL* v.2.4.1 (Schrödinger, LLC) and VMD v.1.9.3. ^28^

## Data availability

Protein Data Bank: The crystal structure of *Pa*FabB C161A with the PDB Id 7PPS, https://doi.org/10.2210/pdb7PPS/pdb.
