# Supplementary material for: Crystal structure of Pseudomonas aeruginosa FabB C161A, a template for structure-based design for new antibiotics
Source: F1000Res. 2022 Jan 10;10:Chem Inf Sci-1102. Originally published 2021 Nov 1. [Version 2] doi: 10.12688/f1000research.74018.2 (PMC8804906; doi:10.12688/f1000research.74018.2)
Supplement: Peer review history

# Peer review: Reviewer response for version 2

**DOI:** 10.5256/f1000research.119244.r119242

**Published:** 2022-01-31

**Authors:** Bjarte A. Lund

**Affiliations:** 1 Hylleraas Centre for Quantum Molecular Sciences, Department of Chemistry, UiT The Arctic University of Norway, Tromsø, Norway

The article has been revised to my satisfaction. No further comments

Is the work clearly and accurately presented and does it cite the current literature?

Partly

If applicable, is the statistical analysis and its interpretation appropriate?

Not applicable

Are all the source data underlying the results available to ensure full reproducibility?

Yes

Is the study design appropriate and is the work technically sound?

Yes

Are the conclusions drawn adequately supported by the results?

Yes

Are sufficient details of methods and analysis provided to allow replication by others?

Yes

Reviewer Expertise:

Biochemistry, structural biology, enzymology,

I confirm that I have read this submission and believe that I have an appropriate level of expertise to confirm that it is of an acceptable scientific standard.

# Peer review: Reviewer response for version 2

**DOI:** 10.5256/f1000research.119244.r119243

**Published:** 2022-01-12

**Authors:** Klaus Reuter

**Affiliations:** 1 Institut für Pharmazeutische Chemie, Philipps-Universität Marburg, Marburg, Germany

All mistakes have been corrected and all concerns have been addressed. The paper may now be indexed as it is, apart from one further correction that has to be done:

- page 10, penultimate line: replace "... selective of dual ..." with "... selective or dual ...".

Is the work clearly and accurately presented and does it cite the current literature?

Partly

If applicable, is the statistical analysis and its interpretation appropriate?

Not applicable

Are all the source data underlying the results available to ensure full reproducibility?

Yes

Is the study design appropriate and is the work technically sound?

Yes

Are the conclusions drawn adequately supported by the results?

Yes

Are sufficient details of methods and analysis provided to allow replication by others?

Yes

Reviewer Expertise:

protein crystallography; enzymology

I confirm that I have read this submission and believe that I have an appropriate level of expertise to confirm that it is of an acceptable scientific standard.

# Peer review: Reviewer response for version 1

**DOI:** 10.5256/f1000research.77724.r101462

**Published:** 2021-12-06

**Authors:** Klaus Reuter

**Affiliations:** 1 Institut für Pharmazeutische Chemie, Philipps-Universität Marburg, Marburg, Germany

First of all: Apart from the fact that I do not insist on CC* or CC1/2 values in Table 2, I do absolutely agree with every single concern of the 1 ^st^ reviewer making it unnecessary to restate all these points.

**Further (minor) issues:**

- Page 3, Introduction, 1 ^st^ paragraph, line 3: Replace “drug-resistant infections” with “infections caused by drug-resistant pathogens”.
- Page 3, Introduction, 2 ^nd ^paragraph, line 4: Replace “w.t. enzyme” with “apo-enzyme”.
- Page 3, Figure 1 (B): In the first partial reaction of the FabF/B-catalysed reaction, the authors indicate the incoming HS-FabF/B (above the reaction arrow) but forget to indicate the leaving HS-ACP.
- Page 4, Results/Protein expression and purification, 2 ^nd ^paragraph, line 2: Replace “… FabB with a cleaved His-tag …” with“… FabB lacking the His-tag …”.
- Page 4, Results/Crystallization of *Pa*FabB C161A, 1 ^st ^paragraph, line 2: Replace “cleaved FabB” with “FabB lacking the His-tag”.
- Page 5, Results/Crystal structure of *Pa*FabB C161A, 1 ^st ^paragraph, line 3: rmsd values for superimposed structures are given with three significant digits, respectively, which suggests inappropriately high accuracy. 0.424 Å should be replaced by 0.42 Å and 0.843 Å by 0.84 Å.
- Page 5, Results/Crystal structure of *Pa*FabB C161A, 1 ^st ^paragraph, line 5: Replace “… from both *Vc*FabB as well as the ones from *Pa*FabB …” with“… from both *Vc*FabB and *Pa*FabB …”.
- Page 6, Results/Active site and differences to *Pa*FabF, 1 ^st ^paragraph, line 1: The 1 ^st^ sentence of this paragraph has to be reformulated. It does not make sense in its present form.
- Page 7, Table 2: Again, several values are given with inappropriately high accuracy. Multiplicity: 6.84 (6.56) should be replaced by 6.8 (6.6); I/sig(I): 19.11 (3.45) should be replaced by 19.1 (3.5); Ramachandran plot, residues in (%): (96.02%) should be replaced by (96.0%) and (3.98%) by (4.0%).
- Page 8, Results/Active site and differences to *Pa*FabF, 1 ^st ^paragraph, line 1: Replace “w.t.” with “apo-”.
- Page 8, Results/Active site and differences to *Pa*FabF, 1 ^st ^paragraph, line 2: Replace “When mutating …” with “Upon the mutation of …”.
- Page 8, Results/Active site and differences to *Pa*FabF, 1 ^st ^paragraph, line 8: Replace “… is mutated to Val268 …” with “… is replaced by Val268 …” or “… corresponds to Val268 …”.
- Figure 5: It is not immediately obvious if the figure shows the homodimer or a single subunit only. This information should be given. If the complete dimer is shown (which I presume) it would make sense to present its subunits in different colours or shades of colour. The same applies to subsequent figures.

**Major concern:**

- The many figures showing FabB or the superpositions of different FabB/F enzymes are not very meaningful. FabB and FabF differ in substrate specificity for the fatty acid chain. The authors should definitely discuss the reasons for the respective substrate specificity by means of available structures.
- I wish the authors had explained why the C161A mutation leads to the open conformation of the active site (see reviewer 1).

In summary, the manuscript is scientifically sound and significant, although somewhat greater effort should have been put into its presentation.

Is the work clearly and accurately presented and does it cite the current literature?

Partly

If applicable, is the statistical analysis and its interpretation appropriate?

Not applicable

Are all the source data underlying the results available to ensure full reproducibility?

Yes

Is the study design appropriate and is the work technically sound?

Yes

Are the conclusions drawn adequately supported by the results?

Yes

Are sufficient details of methods and analysis provided to allow replication by others?

Yes

Reviewer Expertise:

protein crystallography; enzymology

I confirm that I have read this submission and believe that I have an appropriate level of expertise to confirm that it is of an acceptable scientific standard, however I have significant reservations, as outlined above.

# Response

**Published:** 2021-12-22

**Authors:** Ruth Brenk

**Affiliations:** University of Bergen, Norway

Thank you Klaus for reviewing our paper and for very constructive feedback!

 Below follows a point-to-point reply to all issues that you have raised.

  

- First of all: Apart from the fact that I do not insist on CC* or CC1/2 values in Table 2, I do absolutely agree with every single concern of the 1 ^st^ reviewer making it unnecessary to restate all these points.

We have addressed all points that Bjarte Lund has raised. Please refer to the reply to his comments to see what we have changed.

  

- Page 3, Introduction, 1 ^st^ paragraph, line 3: Replace “drug-resistant infections” with “infections caused by drug-resistant pathogens”.

Done

- Page 3, Introduction, 2 ^nd ^paragraph, line 4: Replace “w.t. enzyme” with “apo-enzyme”. 

Done

- Page 3, Figure 1 (B): In the first partial reaction of the FabF/B-catalysed reaction, the authors indicate the incoming HS-FabF/B (above the reaction arrow) but forget to indicate the leaving HS-ACP.

Corrected as suggested 

- Page 4, Results/Protein expression and purification, 2 ^nd ^paragraph, line 2: Replace “… FabB with a cleaved His-tag …” with“… FabB lacking the His-tag …”.

Done

- Page 4, Results/Crystallization of *Pa*FabB C161A, 1 ^st ^paragraph, line 2: Replace “cleaved FabB” with “FabB lacking the His-tag”.

Done

- Page 5, Results/Crystal structure of *Pa*FabB C161A, 1 ^st ^paragraph, line 3: rmsd values for superimposed structures are given with three significant digits, respectively, which suggests inappropriately high accuracy. 0.424 Å should be replaced by 0.42 Å and 0.843 Å by 0.84 Å.

Done

- Page 5, Results/Crystal structure of *Pa*FabB C161A, 1 ^st ^paragraph, line 5: Replace “… from both *Vc*FabB as well as the ones from *Pa*FabB …” with“… from both *Vc*FabB and *Pa*FabB …”.

Done

- Page 6, Results/Active site and differences to *Pa*FabF, 1 ^st ^paragraph, line 1: The 1 ^st^ sentence of this paragraph has to be reformulated. It does not make sense in its present form.

This sentence reads now: “ *The highly conserved Phe400/391 (numbering based on PaFabF/PaFabB) in the malonyl-CoA binding site was previously identified to play a pivotal role in substrate specificity and ligand binding, as this residue adopts different conformations in the apo and the intermediate-binding state*.”

- Page 7, Table 2: Again, several values are given with inappropriately high accuracy. Multiplicity: 6.84 (6.56) should be replaced by 6.8 (6.6); I/sig(I): 19.11 (3.45) should be replaced by 19.1 (3.5); Ramachandran plot, residues in (%): (96.02%) should be replaced by (96.0%) and (3.98%) by (4.0%).

Done 

- Page 8, Results/Active site and differences to *Pa*FabF, 1 ^st ^paragraph, line 1: Replace “w.t.” with “apo-”.

Done 

- Page 8, Results/Active site and differences to *Pa*FabF, 1 ^st ^paragraph, line 2: Replace “When mutating …” with “Upon the mutation of …”.

Done 

- Page 8, Results/Active site and differences to *Pa*FabF, 1 ^st ^paragraph, line 8: Replace “… is mutated to Val268 …” with “… is replaced by Val268 …” or “… corresponds to Val268 …”.

Done 

- Figure 5: It is not immediately obvious if the figure shows the homodimer or a single subunit only. This information should be given. If the complete dimer is shown (which I presume) it would make sense to present its subunits in different colours or shades of colour. The same applies to subsequent figures.

We have added the information to the relevant figures (now 6 and 7) and colored the subunits as suggested.

- The many figures showing FabB or the superpositions of different FabB/F enzymes are not very meaningful. FabB and FabF differ in substrate specificity for the fatty acid chain. The authors should definitely discuss the reasons for the respective substrate specificity by means of available structures.

We have inserted a new section to discuss the substrate selectivity with respect to fatty acids “Comparison of fatty acid chain binding channel in and in *Pa*FabB and *Pa*FabF”. In addition, we have deleted the old Figure 5.

- I wish the authors had explained why the C161A mutation leads to the open conformation of the active site (see reviewer 1).

An explanation has been added to what is now the second paragraph in the section “Comparison of malonyl-CoA binding sites in *Pa*FabB and *Pa*FabF”.

 We hope that we have answered all issues that you have raised to your full satisfaction. If not, please let us know what else we should modify.

# Peer review: Reviewer response for version 1

**DOI:** 10.5256/f1000research.77724.r98455

**Published:** 2021-11-15

**Authors:** Bjarte A. Lund

**Affiliations:** 1 Hylleraas Centre for Quantum Molecular Sciences, Department of Chemistry, UiT The Arctic University of Norway, Tromsø, Norway

Yadrykhins'ky *et al. *present an interesting story on the exploration of a new system for the development of new antibiotics. The authors make a convincing case for why fatty acid synthesis is a good target. Furthermore, the authors convey a clear technical step towards the goal by engineering a mutant variant of one enzyme in the pathway which mimics the intermediate step of the enzymatic reaction. An intermediate state that has been found to be susceptible to other inhibitors.

 My objections to this paper are not on the technical and scientific soundness of the work but on its presentation. 

- The introduction is short and does not clearly convey to me as a non-expert how the mutation of a Cys to Gln/Ala would make the enzyme resemble the intermediate state. I need to read all the way until Figure 9 and the Discussion to be informed about the Phe-residue that flips between open/closed conformations. 
- After reading the paper, it is still not clear to me whether Gln was tried, or whether other amino acids could have had the same effect. Perhaps it is not important since Ala apparently worked. 
- In the Abstract, acyl-intermediate could more clearly be written as acyl-enzyme intermediate.
- Results, Protein expression, and purification,  TEV site would more precisely be termed TEV protease cleavage site.
- Results, Crystallization (Figure 2) **C164A/C161A is mixed non-consistently.** I can only assume this is a mix-up with the group's other work with FabF. But it is very confusing to the reader.
- Clearly out of scope to the paper, but it would have been interesting to know if iodine-phasing was tested.
- It is unclear how the resolution cutoff was decided. I/sig(I) is still very high at the highest resolution shell. It would also have been useful if CC* or CC1/2 was presented in Table 2.
- The very short Discussion-segment could have been merged with the Results-section. The discussion does raise the very interesting possibility of dual-target inhibitors that would presumably be of high interest with regards to the emergence of resistance. This could have been emphasized earlier as well.
- Methods, The sonication amplitude appears to lack a unit, presumably %. For the protein concentration determination, the extinction coefficient used should be given. 
- Reference 21 appears to be a mistake. 

All in all, I did appreciate the work and look forward to the continuation of the story.

Is the work clearly and accurately presented and does it cite the current literature?

Partly

If applicable, is the statistical analysis and its interpretation appropriate?

Not applicable

Are all the source data underlying the results available to ensure full reproducibility?

Yes

Is the study design appropriate and is the work technically sound?

Yes

Are the conclusions drawn adequately supported by the results?

Yes

Are sufficient details of methods and analysis provided to allow replication by others?

Yes

Reviewer Expertise:

Biochemistry, structural biology, enzymology,

I confirm that I have read this submission and believe that I have an appropriate level of expertise to confirm that it is of an acceptable scientific standard, however I have significant reservations, as outlined above.

# Response

**Published:** 2021-12-22

**Authors:** Ruth Brenk

**Affiliations:** University of Bergen, Norway

Thank you Bjarte for reviewing our article!

 Here follows a point-to-point reply to the issues that you have raised.

  

- The introduction is short and does not clearly convey to me as a non-expert how the mutation of a Cys to Gln/Ala would make the enzyme resemble the intermediate state. I need to read all the way until Figure 9 and the Discussion to be informed about the Phe-residue that flips between open/closed conformations. 

We have added a new Figure (now Figure 2) together with some explanatory text in the introduction to make this clearer.

- After reading the paper, it is still not clear to me whether Gln was tried, or whether other amino acids could have had the same effect. Perhaps it is not important since Ala apparently worked. 

We have initially tried the Gln variant, but because this was less stable, we decided to work with the Ala variant instead. We have added this explanation to the end of the introduction.

  

- In the Abstract, acyl-intermediate could more clearly be written as acyl-enzyme intermediate.

Done.

- Results, Protein expression, and purification,  TEV site would more precisely be termed TEV protease cleavage site.

Done.

- Results, Crystallization (Figure 2) **C164A/C161A is mixed non-consistently.** I can only assume this is a mix-up with the group's other work with FabF. But it is very confusing to the reader.

This was indeed a mix-up with the number from FabF which has now been corrected (figure legend for what is now Figure 3).

- Clearly out of scope to the paper, but it would have been interesting to know if iodine-phasing was tested.

We agree that this would be interesting, but we have not yet tested this. We might try it in the future.

- It is unclear how the resolution cutoff was decided. I/sig(I) is still very high at the highest resolution shell. It would also have been useful if CC* or CC1/2 was presented in Table 2

The resolution was limited by the distance of the detector. Unfortunately, we had major problems with shipping crystals to the synchrotron this spring and lost two consecutive shipments due to shipping/custom problems. As the work presented in this paper is from a master thesis, there was no time to repeat the measurements, but it looks like the crystals might diffract even beyond 1.3 Å. We have added CC1/2 to Table 2.

- The very short Discussion-segment could have been merged with the Results-section. The discussion does raise the very interesting possibility of dual-target inhibitors that would presumably be of high interest with regards to the emergence of resistance. This could have been emphasized earlier as well.

We have renamed the Results section to Results and Discussion and the old Discussion section to Conclusions. The discussion about dual-target inhibitors for both, the malonyl-CoA binding site and the fatty acid channel (see comments from Klaus Reuter) has been added to what is now the Results and Discussion section.

  

- Methods, The sonication amplitude appears to lack a unit, presumably %. For the protein concentration determination, the extinction coefficient used should be given. 

Done

- Reference 21 appears to be a mistake. 

Deleted.

 We hope that we have answered to all issues that you have raised to your full satisfaction. If not, please let us know what else we should address.
